# Asymmetric Organocatalytic Mannich Reaction in the Synthesis of Hybrid Isoindolinone-Pyrazole and Isoindolinone-Aminal from Functionalized α-Amidosulfone

**DOI:** 10.3390/ijms24065783

**Published:** 2023-03-17

**Authors:** Antonia Di Mola, Felice De Piano, Lorenzo Serusi, Giovanni Pierri, Laura Palombi, Antonio Massa

**Affiliations:** 1 Dipartimento di Chimica e Biologia “A. Zambelli”, Università Degli Studi di Salerno, Via Giovanni Paolo II, 84084 Fisciano, SA, Italy; 2 Dipartimento di Scienze Fisiche e Chimiche, Università dell’Aquila, 10-67100 Coppito, AQ, Italy

**Keywords:** heterocycles, organocatalysis, asymmetric synthesis, hybrid molecules

## Abstract

The investigation of the reactivity of an α-amido sulfone derived from 2-formyl benzoate under organocatalytic conditions in the presence of acetylacetone allowed the synthesis of a new heterocyclic hybrid isoindolinone-pyrazole with high enantiomeric excess. Dibenzylamine was also used as a nucleophile to afford an isoindolinone with aminal substituent in 3-position in suitable selectivity. The use of Takemoto’s bifunctional organocatalyst not only led to observed enantioselectivity but was also important in accomplishing the cyclization step in both cases. Notably, this catalytic system proved to be particularly effective in comparison to widely used phase transfer catalysts.

## 1. Introduction

**Figure 1:**
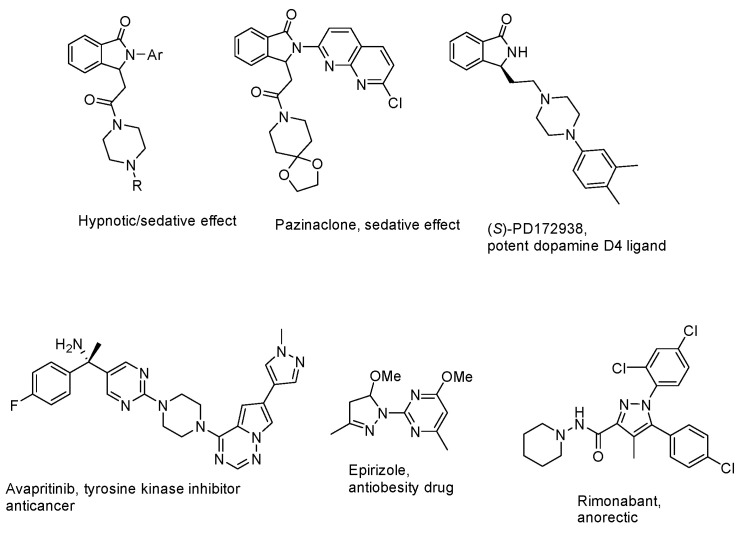
Relevant bioactive isoindolinone (first row) and pyrazole (second row) hybrids.

The value of some classes of compounds is often associated with the structural diversity and the presence of different functionalities and moieties. This feature is relevant in classes of heterocycles such as isoindolinones [1,2,3,4,5,6,7,8,9] and pyrazoles [10,11,12], whose biological activities are tuned by the linking of additional nitrogen-containing substituents or heterocyclic groups such as piperazines, piperidines, and so on (Figure 1).

Therefore, the development of new synthetic methods to furnish new hybrid molecules is of paramount importance, even though additional difficulties can be encountered, especially when new stereocenters are formed [13]. In fact, asymmetric construction of the isoindolinone ring is a challenging research field, and many groups are involved [13]. It is worth noting that enantiopure isoindolinones have been reported to show enhanced biological properties, but often inefficient resolution of racemic mixtures or the use of chiral auxiliaries have been utilized to achieve this goal [13]. In this context, our group dedicated many efforts to the asymmetric synthesis of 3-substituted [14,15,16] and 3,3-disubstituted isoindolinones, [17,18] developing new cascade-type reactions often employing organocatalytic systems and producing several new enantioenriched products. Some of the obtained isoindolinones were also used in the total synthesis of biologically relevant compounds [5,15,18]. The reasons for the success of the developed methods also rely on the rational design and synthesis of suitable starting materials. Recently, readily available α-amido sulfones derived from 2-formyl benzoates revealed effective starting materials in the asymmetric synthesis of 3-nitromethyl isoindolinones, reaching enantioselectivities up to 98% ee and suitable yields in the process of asymmetric aza-Mannich/lactamization reaction [16]. Takemoto’s neutral bifunctional organocatalyst [19] was not only essential to achieving high enantioselectivity but was also crucial for catalyzing the cyclization step of the process [13].

The easy access and high stability of these N-carbamoyl-α-amidosulfones gives several practical advantages avoiding, first of all, the use of the respective preformed imines, which were difficult to isolate, while the formation of the imines from α-amidosulfones can be easily carried out *in situ* in the presence of an inorganic base [20,21]. This approach is explored in asymmetric catalysis for the synthesis of functionalized chiral amines in nitro-Mannich reactions [16,20,21], while different nucleophiles [22,23] as dicarbonyl compounds [22] or amines are less investigated or not investigated at all. In these asymmetric transformations, chiral ammonium salts, employed as phase transfer catalysts, play a fundamental role in attaining high enantioselectivity [20,21,23], while neutral chiral organocatalysts have been less investigated [16,22]. The presence of easily removable *N*-Boc or *N*-Cbz groups is a further synthetic advantage of these methods [16,20,21].

Given the efficiency of the method in the synthesis of 3-nitromethyl isoindolinones [16] and the utility of the target compounds, the application of this methodology to different classes of nucleophiles is of paramount importance to attain new, differently decorated derivatives.

Therefore, in the present investigation, we describe the asymmetric synthesis of a new heterocyclic hybrid molecule as a consequence of the use of acetylacetone as a nucleophile in an asymmetric reaction with *N*-carbamoyl-α-amidosulfone derived from 2-formylbenzoate in the presence of Takemoto’s bifunctional organocatalyst. The investigation of the reaction mechanism led to the development of a new synthetic route leading to the obtaining of a new hybrid isoindolinone-pyrazole in high enantioselectivity. The use of dibenzylamine as a nucleophile was also investigated for comparison. In both cases, Takemoto’s bifunctional organocatalyst was more effective than benchmark chiral phase transfer catalysts.

## 2. Results and Discussion

### 2.1. Investigation of the Reactivity of the α-Amido Sulfone 1 under Asymmetric and Achiral Conditions

**Figure 2:**
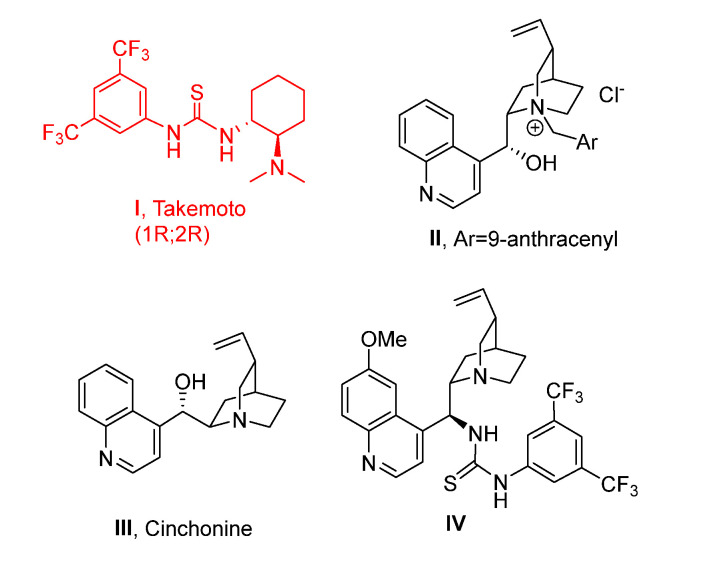
Organocatalysts investigated in this methodology.

In the first set of experiments, α-amido sulfone **1** was reacted with acetylacetone in the presence of chiral neutral bifunctional organocatalysts and an ammonium salt in combination with an inorganic base (Figure 2 and Table 1).

We soon realized that the additional step is rapid, even below ambient temperature, while the cyclization is slow at room temperature, probably because of the less nucleophilicity of a NH belonging to a carbamate group. In the presence of Takemoto’s catalyst **I**, even after 72 h of stirring at room temperature, we recovered acyclic product **2** with moderate enantioselectivity in DCM (Table 1, Entry 1). Enantiomeric excess improved by decreasing the temperature to −40 °C, although the moderate yield was obtained probably due to some decomposition of **2** after long stirring at rt. (Entries 2 and 3). Therefore, we repeated the experiment performing the purification at the end of the addition step at −40 °C, confirming the high enantioselectivity but with improved yield (Entry 4). Focusing only on the addition step, other catalytic systems were also tested for comparison as the PTC chiral ammonium salt **II** derived from quinine (Entry 5), cinchonine **III** (Entry 6), or *epi*-quinine bifunctional organocatalyst **IV** (Entry 7). Good enantioselectivity was also obtained with chiral ammonium salt **II** (Entry 5), but Takemoto’s catalyst was the best performing one affording **2** with 88% ee.

At this point, we thought that the increase in the temperature after the addition step could favor lactamization. To achieve this goal, we had to use a higher boiling solvent as 1,2-DCE in a mixture with DCM since 1,2-DCE has an unsuitable melting point of −35 °C, and DCM has an unsuitable boiling point of 40 °C. We first repeated the asymmetric addition step, confirming the goodness of the used mixture of solvents leading to similar results in terms of yield and enantioselectivity (Entry 8). Then, in a second experiment, we first performed the addition step at −40 °C until the reaction was complete, as checked by TLC, and then the reaction mixture was warmed up to 60 °C. This experiment was successful since the target cyclic isoindolinone was obtained in suitable yield but, unfortunately, as a racemate (Entry 9).

The reaction was also investigated under achiral conditions in the presence of K_2_CO_3_ only (Table 2). In all the experiments, we recovered the acyclic product **2**, even after prolonged heating at 60 °C and in different solvents such as toluene, DCM, or acetonitrile, confirming the importance of using Takemoto’s catalyst **I** for the cyclization process. On the other hand, the addition process appears to be slow at −40 °C (Entries 1 and 2), explaining the high enantioselectivity (see Table 1) because of the lack of the background achiral reaction at low temperature.

### 2.2. Investigation of the Cyclization of Intermediate 2

**Figure 3:**
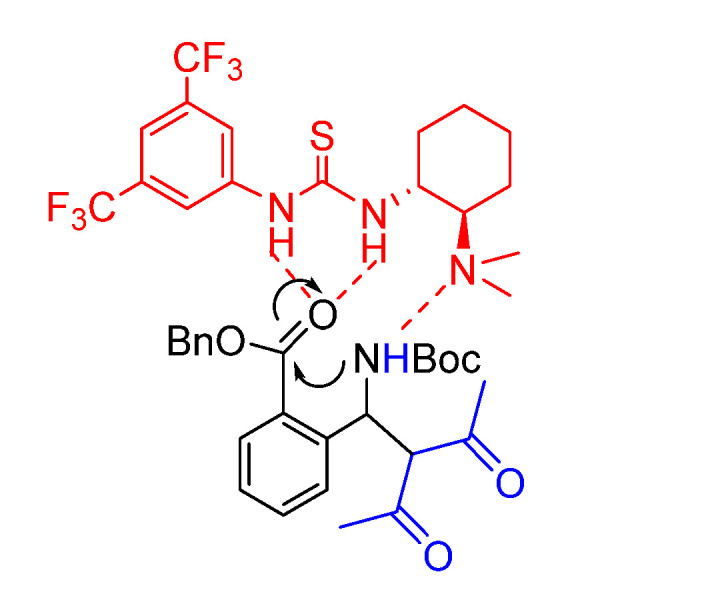
Proposed TS for catalyst-mediated cyclization.

Cyclization was investigated directly on the purified enantioenriched acyclic product **2**. Several cyclization conditions were tested (Table 3). Reaction performed at 60 °C in the presence of K_2_CO_3_ only provided decomposition products, confirming the hypothesis that Takemoto’s catalyst is important for promoting lactamization (Entries 1 and 2), probably with a mechanism as described in Figure 3. When a combination of K_2_CO_3_ and Takemoto’s catalyst was used, we were able to obtain the cyclic product, but unfortunately, as a racemate (Entries 3–7). The use of a catalytic amount of both Takemoto’s catalyst and K_2_CO_3_ at 40 °C was effective as well, but again racemization was observed (Entry 6). This experiment was repeated and stopped at about 50% conversion (Entry 7). Again, the cyclic product was racemic, while the unreacted acyclic intermediate maintained 88% ee.

**Scheme 1:**
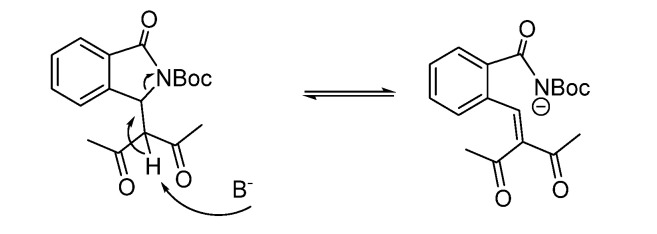
Proposed racemization process.

All these experiments point out that racemization may occur on the cyclic product, probably via a retro aza-Michael reaction under basic conditions, as depicted in Scheme 1. Removing the acidic proton of acetylacetone moiety should prevent racemization.

### 2.3. Asymmetric Synthesis of Isoindolinone-Pyrazole Hybrid

**Scheme 2:**
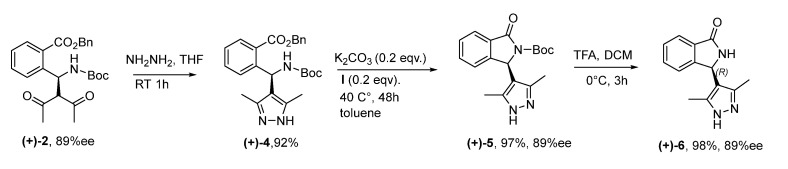
Asymmetric synthesis of isoindolinone-pyrazole hybrid.

**Figure 4:**
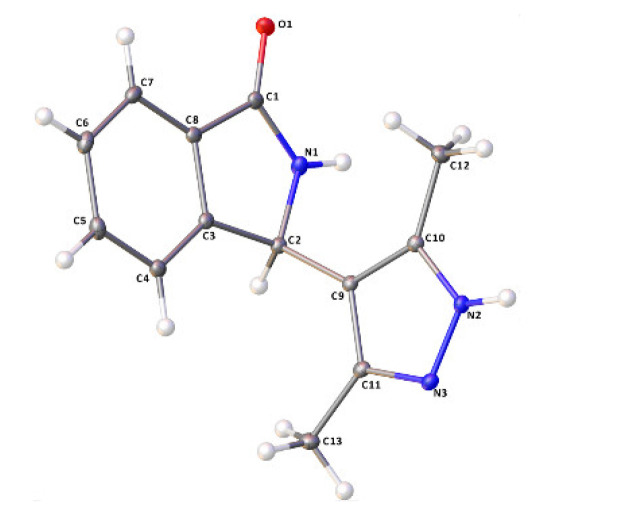
ORTEP drawings for compound (+)-**6** (CCDC 2246867).

In order to confirm this hypothesis and overcome the racemization issue, our attention focused on the use of hydrazine, which should react selectively with acetylacetone to provide pyrazole ring, [24] a moiety also found in a very large number of bioactive compounds, [10,11,12] often combined with other heterocycles [12]. Therefore, the enantioenriched acyclic product **2** was treated with hydrazine under the conditions shown in Scheme 2, leading nicely to the pyrazole derivative **4** in a very high yield. Then, it was subjected to cyclization, under the conditions of Table 3, Entry 6, nicely furnishing the *N*-Boc protected isoindolinone **5**. This derivative was, then, deprotected in the presence of TFA, without any decrease in enantiopurity as determined by HPLC on chiral stationary phase leading to NH-free hybrid isoindolinone-pyrazole **6** with unchanged enantiopurity of 89% ee. Absolute configuration (AC) of (+)-**6** was found to be (*R*) by X-ray analysis performed on a suitable single crystal (Figure 4 and Figure S1 and Scheme 2). This finding also allowed us to assign the AC of all the previous intermediates and, therefore, of (+)-**2,** which was obtained with catalyst (*R,R*)-**I**. The sequence of reactions was also performed by readily available (*S,S*)-**I**, leading to similar results in terms of yields and enantiomeric purity for the opposite enantiomer (*S*)-**2**, demonstrating the goodness of our hypothesis and feasibility of the adopted strategy to prevent racemization.

**Scheme 3:**
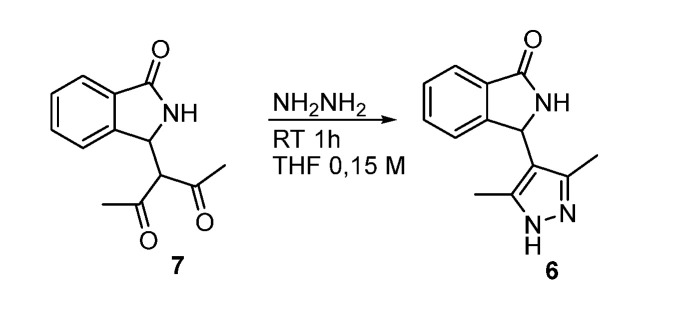
Synthesis of racemic isoindolinone-pyrazole hybrid.

The racemic hybrid compound was alternatively obtained by reaction of 3-acetylacetone isoindolinone [25] **7** with hydrazine (Scheme 3, see Supplementary Materials for further experimental details). On the other hand, when both **2** and **7** were treated with phenylhydrazine, we recovered the starting materials unreacted.

### 2.4. Reaction with Dibenzylamine (DBA)

The reactivity of **1** was next investigated in the presence of the secondary amine DBA with the aim of producing a 3-substituted isoindolinone with an aminal functionality. Several catalytic systems were tested as well (Table 4 and Figure 2).

As noted from the data reported in Table 4, the best results were obtained again in the presence of Takemoto’s catalyst **I** at −20 °C in toluene (Entry 7), while other catalytic systems such as the PTC **II** (Entry 4), cinchonine **III** (Entry 5) and the thiourea-*epi*-quinine **IV** (Entry 6) were less effective both in the preliminary screening at RT as well as at −20 °C (Entry 8). In this investigation, both (*R,R*)-**I** and (*S,S*)-**I** were used, leading the opposite enantiomers of **9** as analyzed by HPLC on chiral stationary phase. Interestingly, control experiments performed in the absence of K_2_CO_3_ and with 2 eqv of DBA highlighted that the inorganic base is essential to obtain satisfactory yield and enantioselectivity (Entries 1 and 10). Other conditions were also investigated as different solvents (Entries 3 and 9), lower temperature (Entry 11), and other catalytic systems such as Maruoka’s catalyst, a squaramide bifunctional organocatalyst and a PTC derived from (*1R,2R*)-diaminocyclohexane. All these catalysts were ineffective as well (for brevity, these data were reported in the Supplementary Materials, Table S1).

Also, in the presence of this nucleophile, the cyclization proved to be slow below RT but faster than previously observed with intermediate **2**. In fact, further stirring the reaction mixture at RT was enough to obtain the expected cyclic product, usually in very suitable yields.

## 3. Materials and Methods

### General Information

Unless otherwise noted, all chemicals, reagents, and solvents for the performed reactions are commercially available. α-Amidosulfone **1** was prepared according to a literature procedure [16]. All the reactions were monitored by thin layer chromatography (TLC) on precoated silica gel plates (0.25 mm) and visualized by fluorescence quenching at 254 nm. Flash chromatography was carried out using silica gel 60 (70–230 mesh, Merck, Darmstadt, Germany). Yields are given for isolated products showing one spot on a TLC plate, and no impurities were detectable in the NMR spectrum. The NMR spectra were recorded on Bruker DRX 600, 400, and 300 MHz spectrometers (600 MHz, ^1^H, 150 MHz, ^13^C; 400 MHz, ^1^H, 100.6 MHz; ^13^C, 300 MHz, ^1^H, 75.5 MHz, ^13^C, 250 MHz, ^1^H, 62.5 MHz, ^13^C). Internal reference was set to the residual solvent signals (δ_H_ 7.26 ppm, δ_C_ 77.16 ppm for CDCl_3_). The ^13^C NMR spectra were recorded under broad-band proton decoupling. Spectra are reported only for unknown compounds. The following abbreviations are used to indicate the multiplicity in NMR spectra: s-singlet, d-doublet, t-triplet, q-quartet, dd-doublet of doublets, m-multiplet, brs-broad signal. Coupling constants (J) are quoted in Hertz. High-resolution mass spectra (HRMS) were acquired using a Bruker SolariX XR Fourier transform ion cyclotron resonance mass spectrometer (Bruker Daltonik GmbH, Bremen, Germany) equipped with a 7T refrigerated actively shielded superconducting magnet. For ionization of the samples, electrospray ionization (ESI) or MALDI was applied. IR spectra were recorded on an IR Bruker Vertex 70 v spectrometer. Polarimeter Jasco P-2000 (Tokyo, Japan). The crystal was measured on a Bruker D8 QUEST diffractometer equipped with a PHOTON detector using Cu-*Kα* radiation (λ= 1.54178 Å). Data indexing (Table S2), reduction and absorption correction were performed using APEX3 software suite, version 2015.5-2 [26,27,28]. The structure was solved using SHELXS [29] and refined by means of full matrix least-squares based on *F*^2^ using the program SHELXL [30] and OLEX2 [31] as a GUI. The Flack parameter was defined accordingly to ref [32].

**Benzyl 2-(2-acetyl-1-((tert-butoxycarbonyl)amino)-3-oxobutyl)benzoate (*R*)-2.** In an ACE tube, **1** (120 mg, 0.24 mmol, eqv.), K_2_CO_3_ (66 mg, 2 eqv.), acetylacetone (66 μL, 2 eqv.), (*R,R*)-Takemoto catalyst **I** (20 mol%) were stirred at −40 °C in dichoroethane (1.2 mL)/dichloromethane (800 μL) until the starting material was completely consumed (24 h). The inorganic salt was filtered off, and the mixture was directly purified by flash chromatography on silica gel (hexane/ethyl acetate from 9/1 to 8/2), recovering a pale oil. Yield: 80% (84 mg). Spectroscopic data were found in agreement with the racemate. HRMS (MALDI): *m/z* calcd for [C_25_H_29_NO_6_ + K]^+^: 478.16265; found: 478.16545. [α]_D_^20^: +80.9 (c 1, CHCl_3_). Enantiomeric excess was determined on chiral HPLC Chiral column IE-3 Hexane/Isopropanol 80/20 0.6 mL/min, λ 220 and 254 nm. Ee: 89%, t_1_: 24.3 min and t_2_: 27.4 min.

**tert-butyl 1-(2,4-dioxopentan-3-yl)-3-oxoisoindoline-2-carboxylate 3**. From the open intermediate, no reaction occurs, warming the mixture to 50 °C for 2 days (DCE/DCM 0.1 M), using only potassium carbonate (2 eqv.). The addition of racemic Takemoto catalyst (20 mol%) let us recover the product in almost quantitative yield.

**Benzyl 2-(((tert-butoxycarbonyl)amino)(3,5-dimethyl-1H-pyrazol-4-yl)methyl)benzoate (*R*)-4.** To a solution of enantioenriched intermediate (+)-**2** (40 mg, 0.12 mmol, 1 eqv.) in THF (600 µL) hydrazine hydrate 80% was added (8 µL, 1.1 eqv.) and the mixture was stirred for 1 h. The solvent was removed under reduced pressure, and the crude was purified by flash chromatography on silica gel (Hexane/Ethyl acetate from 6/4 to 3/7), recovering a pale oil. Yield: 92% (48 mg). ^1^HNMR (CDCl_3_, 400 MHz) δ 7.78 (d, *J* = 7.7 Hz, 1H), 7.59 (app dd, *J_1_* = 7.7 Hz, *J_2_* = 1.3 Hz, 1H), 7.49 (dt, *J* = 6.5 Hz, 1H), 7.41–7.23 (m, 7H), 6.63 (d, *J* = 7.7 Hz, 1H), 6.16 (br s, 1H), 5.10 (s, 2H), 1.87 (s, 6H), 1.44 (s, 9H). ^13^CNMR (CDCl_3_, 100 MHz) δ 167.4, 155.0, 142.9, 142.4, 135.9, 131.6, 130.8, 130.1, 128.7, 128.2, 128.1, 127.4, 127.2, 115.8, 79.8, 66.8, 48.4, 28.5, 11.5. IR (neat): 3200, 2940, 2880, 1689, 1672,1656 cm^−1^. HRMS (MALDI): *m/z* calcd for [C_25_H_29_N_3_O_4_ + K]^+^: 474.17896; found: 474.17335. [α]_D_^20^: +12.6 (c 0.6, CHCl_3_). Enantiomeric excess was determined after cyclization.

**tert-butyl 1-(3,5-dimethyl-1H-pyrazol-4-yl)-3-oxoisoindoline-2-carboxylate (*R*)-5.** A solution of compound (+)-**4** (50 mg, 0.11 mmol, 1 eqv.) in toluene (1.1 mL), potassium carbonate (4 mg, 20 mol%), and (*R*,*R*)-Takemoto catalyst **I** (20 mol%) was warmed to 40 °C for 48 h, until starting material was consumed. The crude was purified by flash chromatography on silica gel (hexane/ethyl acetate from 6/4 to 3/7), recovering an amorphous solid. Yield: 97% (35 mg). ^1^HNMR (CDCl_3_, 300 MHz) δ 7.93 (d, *J* = 7.5 Hz, 1H), 7.59 (t, *J* = 7.5 Hz, 1H), 7.49 (t, *J* = 7.5 Hz, 1H), 7.17 (d, *J* = 7.5 Hz, 1H), 5.98 (s, 1H), 5.13 (br s, 1H), 1.45 (s, 15H). ^13^CNMR (CDCl_3_, 100 MHz) δ 166.9, 150.1, 145.1, 143.0, 134.1, 130.4, 128.9, 124.7, 123.4, 112.3, 83.1, 55.9, 28.1, 11.1. IR (KBr disc): 3200, 2940, 2880, 1670, 1656 cm^−1^. HRMS (MALDI): *m/z* calcd for [C_18_H_21_N_3_O_3_ + H]^+^: 328.16557; found: 328.16678. [α]_D_^20^: +35.7 (c 1, CHCl_3_). Enantiomeric excess was determined by chiral HPLC. Chiral colunm OD Hexane/Isopropanol 80/20 0.6 mL/min, λ 220 and 254 nm. Ee: 89%, t_1_: 9.1 min and t_2_: 10.9 min.

**3-(3,5-dimethyl-1H-pyrazol-4-yl)isoindolin-1-one (*R*)-6.** At 0 °C, to a chilled solution of compound (+)-**5** (30 mg, 0.09 mmol, 1 equiv.) in dichoromethane (800 µL) was added trifluoroacetic acid (TFA, 130 µL) and the mixture was stirred for 3 h. The suspension was diluted with dichoromethane, basified with NaOH 1N, and washed with brine. The crude obtained after evaporation of the solvent was purified by flash chromatography on silica gel (ethyl acetate/methanol 95/5), recovering an amorphous solid. Yield: 98% (11 mg). ^1^HNMR (MeOD, 400 MHz) δ 7.83 (d, *J* = 7.4 Hz, 1H), 7.61 (t, *J* = 7.4 Hz, 1H), 7.53 (t, *J* = 7.4 Hz, 1H), 7.31 (d, *J* = 7.04 Hz, 1H), 5.76 (s, 1H), 1.96 (s, 6H). ^13^CNMR (MeOD, 100 MHz) δ 171.4, 147.8, 132.2, 131.7, 127.9, 123.1, 122.7, 110.6, 52.1, 9.1. IR (KBr disc): 3200, 2940, 2880, 1656 cm^−1^. HRMS (MALDI): *m/z* calcd for [C_13_H_13_NO + H]^+^: 228.11314; found: 228.11156. [α]_D_^20^: +19.4 (c 0.3, CHCl_3_). Enantiomeric excess was determined by chiral HPLC. Chiral column OD 60/40 0.6 mL/min, λ 220 and 254 nm. Ee: 89%, t_1_: 5.4 min and t_2_: 7.6 min.

**tert-butyl 1-(dibenzylamino)-3-oxoisoindoline-2-carboxylate 9**. To a solution of α-amido sulfone **1** (40 mg, 0.08 mmol, 1.0 equiv.) in toluene (1.6 mL), dibenzylamine (1.5 equiv., 0.12 mmol), K_2_CO_3_ (55 mg, 0.4 mmol, 5 equiv.) and (*S*, *S*) -Takemoto catalyst (20 mol%) were added, and the reaction mixture was stirred at -20 °C, until the starting material completely disappear. Then the reaction was allowed to stir at room temperature until the intermediate was fully converted into the cyclic product. Purification on silica gel (Hexane/ Ethyl acetate, 5:1) afforded the resulting cyclic product as an oil. Yield: 88% (30 mg.).^1^H NMR (300 MHz, CDCl_3_) δ 7.78 (d, *J* = 7.28 Hz, 1H), 7.60–7.55 (m, 2H), 7.47–7.20 (m, 12H), 6.13 (s, 1H), 3.84 (d, *J* = 12.5 Hz, 2H), 3.66 (d, *J* = 12.5 Hz, 2H), 1.65 (s, 9H). ^13^CNMR (100 MHz, CDCl_3_) δ 166.3, 151.9, 143.6, 139.2, 133.9, 131.2, 129.7, 129.3, 129.2, 128.7, 128.6, 128.5, 128.4, 127.3, 124.4, 124.2, 83.3, 76.4, 53.2, 28.3. HRMS (MALDI): *m/z* calcd for [C_27_H_28_N_2_O_3_ + H]^+^: 429.2173; found: 429.2190. [α]_D_^18^: = +88 (c = 0.8, CHCl_3_). Enantiomeric excess was determined by chiral HPLC. Chiral column OD-H, hexane/isopropanol, 95:5, 0.6 mL/min, λ 220 and 254 nm. Ee: 63%, t_1_: 9.1 min and t_2_: 10.0 min.

## 4. Conclusions

In this work, we have investigated the Mannich reaction of an α-amido sulfone derived from 2-formyl benzoate under organocatalytic conditions in the presence of acetylacetone and dibenzylamine as nucleophiles. The investigation of the reactivity of acetylacetone derivative allowed to synthesize a new heterocyclic hybrid molecule formed by an isoindolinone substituted in 3-position with a pyrazole ring in high enantioselectivity of 89% ee and an overall yield of about 70% in 4 synthetic steps. Moderate enantioselectivity was observed using dibenzylamine as a nucleophile leading to a 3-substituted isoindolinone with an aminal functionality. In both cases, Takemoto’s bifunctional organocatalyst proved to be more effective than other catalytic systems as phase transfer catalysts (PTC). In this investigation, the bifunctional nature of the used catalyst also favored lactamization, as confirmed by a series of control experiments.

## Figures and Tables

**Table 1:**
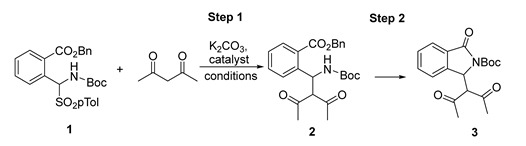
Reaction conditions investigation of the two-step process with chiral organocatalysts.

Entry	Cat (20 mol%)	Solvent	Time (h) Step 1/Step 2	T (°C) Step 1/Step 2	2 or 3, Yield (%) ^1^	2 or 3, ee (%) ^2^
1	**I**	DCM	72	rt	**2,** 56	**2,** 40
2	**I**	toluene	24/72	−40/rt	**2,** 59	**2,** 69
3	**I**	DCM	24/72	−40/rt	**2,** 56	**2,** 88
4	**I**	DCM	24/0	−40	**2,** 80	**2,** 88
5	**II**	DCM	50/0	−40	**2,** 82	**2,** 77
6	**III**	DCM	21/0	−40	**2,** 35	**2,** 40
7	**IV**	DCM	24/0	−40	**2,** 56	**2,** 56
8	**I**	DCE/DCM	24/0	−40	**2,** 80	**2,** 89
9	**I**	DCE/DCM	24/48	−40/60	**3,** 80	**3,** 0

^1^ Isolated yield. ^2^ Determined by HPLC on chiral column.

**Table 2:**
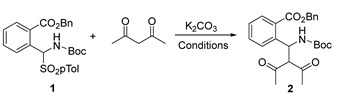
Control experiments under achiral conditions.

Entry	Solvent	T (°C)	Time (h)	Yield (%) ^1^
1	Toluene	−40	30	-
2	DCM	−40	30	-
3	Toluene	rt	30	77
4	Toluene	rt	24	79
5	CH_3_CN	rt	24	35
6	CH_3_CN	rt	69	69
7	DCE	60	20	70
8	toluene	60	20	72

^1^ Isolated yield.

**Table 3:**
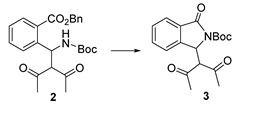
Investigation of the cyclization process.

Entry	Cat. I/K_2_CO_3_ (Eqv/Eqv)	Solvent	T (°C)	Time (h)	Yield (%) ^1^	e.e (%) ^2^
1	0/1	CH_3_CN	60	48	-	
2	0/1	toluene	60	48	-	
3	0.2/1	toluene	60	36 h	86	0
4	0.2/1	DCE	60	24 h	60	0
5	0.2/1	DCE/DCM	60	24 h	94	0
6	0.2/0.2	toluene	40	50 h	90	0
7	0.2/0.2	toluene	40	24 h	43	0

^1^ Isolated yield. ^2^ Determined by HPLC on chiral column.

**Table 4:**
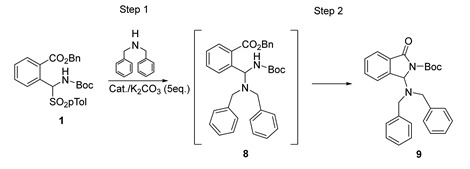
Investigation of asymmetric reaction of **1** with dibenzylamine.

Entry	Cat (20 mol%)	Solvent	Time (h) Step 1/Step 2	T (°C) Step 1/Step 2	9, Yield (%) ^1^	9, ee (%) ^2^
1 ^3^	(*S,S*)-**I**	Toluene	20	RT	-	-
2	(*R,R*)-**I**	Toluene	90	RT	83	-36
3	(*S,S*)-**I**	DCM	144	RT	96	20
4	**II**	Toluene	140	RT	75	rac
5	**III**	Toluene	160	RT	92	rac
6	**IV**	Toluene	72	RT	89	rac
7	(*S,S*)-**I**	Toluene	7/69	−20/RT	88	63
8	**II**	Toluene	8/60	−20/RT	78	−20
9	(*R,R*)-**I**	*m*-Xylene	7/45	−20/RT	88	−54
10^3^	(*R,R*)-**I**	Toluene	7/69	−20/RT	35	−12
11	(*R,R*)-**I**	Toluene	8/115	−40/RT	94	−60

^1^ Isolated yield. ^2^ Determined by HPLC on chiral column. ^3^ Rection performed without K_2_CO_3_ and with 2 eqv of DBA.

## Supplementary Materials

The following supporting information can be downloaded at: https://www.mdpi.com/article/10.3390/ijms24065783/s1.
