# Supplementary material for: Asymmetric Organocatalytic Mannich Reaction in the Synthesis of Hybrid Isoindolinone-Pyrazole and Isoindolinone-Aminal from Functionalized α-Amidosulfone
Source: Int J Mol Sci. 2023 Mar 17;24(6):5783. doi: 10.3390/ijms24065783 (PMC10053823; doi:10.3390/ijms24065783)
Supplement: Supplementary file 1 [file ijms-24-05783-s001.zip › ijms-2246842-supplementary.pdf]

Supporting information

Asymmetric Organocatalytic Mannich Reaction in the Synthesis of Hybrid Isoindolinone-Pyrazole and Isoindolinone-Aminal from Functionalized α-Amidosulfone

Antonia Di Mola [1], Felice De Piano [1], Lorenzo Serusi [1], Giovanni Pierri [1], Laura Palombi [2] and Antonio Massa [1,*]

1 Dipartimento di Chimica e Biologia "A. Zambelli", Università Degli Studi di Salerno, Via Giovanni Paolo II, 84084 Fisciano, SA, Italy; toniadimola@libero.it (A.D.M.); f.depiano6@studenti.unisa.it (F.D.P.); lserusi@unisa.it (L.S.); gpierri@unisa.it (G.P.)
2 Dipartimento di Scienze Fisiche e Chimiche, Università dell'Aquila, 10-67100 Coppito, AQ, Italy; laura.palombi@univaq.it
* Correspondence: amassa@unisa.it

Table of Contents

General information S2

One-pot procedure for the racemic synthesis of compound 3 .... S2

Two steps procedure for the racemic synthesis of compound 3 .... S3

Synthesis of racemic pyrazole derivatives 5 and 6 .... S4

One-pot enantioselective procedure for the synthesis of compound 3 .... S5

Two steps enantioselective procedure for the synthesis of compound 3 and 5 .... S5

Deprotection of isoindolinone 5 .... S7

Reaction with dibenzylamine S7

Crystallographic data of (+)-6 .... S9

References S11

Copies of ^{1}H NMR and ^{13}C NMR spectra of products S12

HPLC traces S18

Experimental section

General information

Unless otherwise noted, all chemicals, reagents and solvents for the performed reactions are commercially available. Amidosulfone was prepared according to a literature procedure.[1] All the reactions were monitored by thin layer chromatography (TLC) on precoated silica gel plates (0.25 mm) and visualized by fluorescence quenching at 254 nm. Flash chromatography was carried out using silica gel 60 (70–230 mesh, Merck, Darmstadt, Germany). Yields are given for isolated products showing one spot on a TLC plate and no impurities were detectable in the NMR spectrum. The NMR spectra were recorded on Bruker DRX 600, 400, and 300 MHz spectrometers (600 MHz, ^{1}H, 150 MHz, ^{13}C; 400 MHz, ^{1}H, 100.6 MHz; ^{13}C, 300 MHz, ^{1}H, 75.5 MHz, ^{13}C, 250 MHz, ^{1}H, 62.5 MHz, ^{13}C). Internal reference was set to the residual solvent signals (δ_H 7.26 ppm, δ_C 77.16 ppm for $CDCl_3$). The ^{13}C NMR spectra were recorded under broad-band proton-decoupling. Spectra are reported only for unknown compounds. The following abbreviations are used to indicate the multiplicity in NMR spectra: s-singlet, d-doublet, t-triplet, q-quartet, dd-doublet of doublets, m-multiplet, brs-broad signal. Coupling constants (J) are quoted in Hertz. High resolution mass spectra (HRMS) were acquired using a Bruker SolariX XR Fourier transform ion cyclotron resonance mass spectrometer (Bruker Daltonik GmbH, Bremen, Germany) equipped with a 7T refrigerated actively-shielded superconducting magnet. For ionization of the samples electrospray ionization (ESI) or MALDI was applied. IR spectra were recorded on a IR Bruker Vertex 70v spectrometer. Polarimeter Jasco P-2000 (Tokio, Japan).

One-pot procedure for the racemic synthesis of compound 3

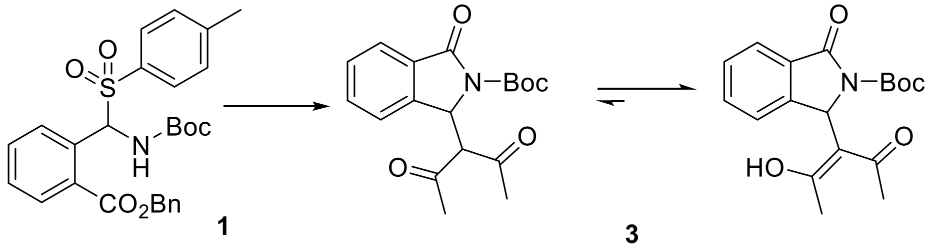

***tert*-butyl 1-(2,4-dioxopentan-3-yl)-3-oxoisoindoline-2-carboxylate 3.** In an ACE tube, **1** (120 mg, 0.24 mmol, equiv.), K_2CO_3 (66 mg, 2 equiv), acetylacetone (66 µL, 2 equiv), racemic Takemoto catalyst (20 mol%) were stirred at room temperature in dichoroethane (1.2 mL)/ dichloromethane (800 µL) until the starting material was completely consumed (16 h). Then, the reaction mixture was warmed to 50-60 °C for 2 days. The inorganic salt was filtered off and the mixture was directly purified by flash chromatography on silica gel (Hexane/Ethyl acetate from 9/1 to 7/3) recovering **3** as white solid. Yield: 88% (70 mg). M.p. 81-82°C. ^{1}HNMR ($CDCl_3$, 300 MHz, enol form) δ 7.94 (d, *J*= 7.4 Hz, 1H), 7.67 (t, *J*= 7.4 Hz, 1H), 7.54 (t, *J*= 7.4 Hz, 1H), 7.34 (d, *J*=

7.4 Hz, 1H), 5.99 (s, 1H, enol form), 2.53 (s, 3H), 1.55 (s, 9H), 1.49 (s, 3H). ^{13}CNMR ($CDCl_3$, 75 MHz, enol form) δ 193.9, 192.3, 166.4, 150.1, 144.6,141.8, 134.4, 134.2, 130.8, 129.2, 125.1, 122.6, 107.8, 83.7, 58.4, 57.9, 31.9, 30.4, 28.2, 23.9, 23.7. IR (KBr disc): 2945, 1684, 1673, 1656 cm^{-1}. HRMS (MALDI): *m/z* calcd for $[C_{18}H_{21}NO_5 + K]^+$: 370.10513; found: 370.10581.

Two steps procedure for the racemic synthesis of compound 3

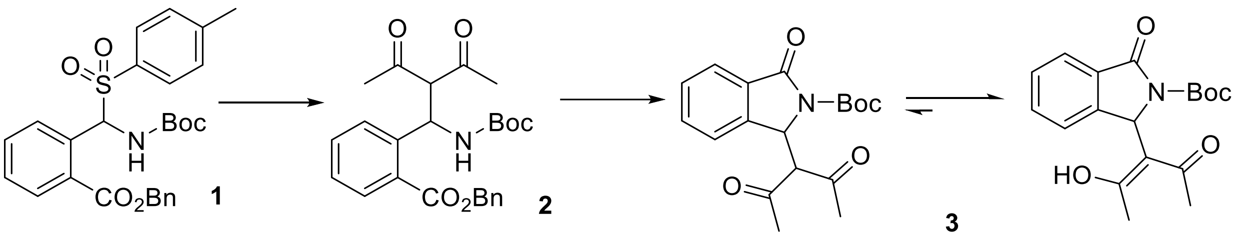

Step 1.

Benzyl 2-(2-acetyl-1-((tert-butoxycarbonyl)amino)-3-oxobutyl)benzoate 2. In an ACE tube, α-amido sulfone **1** (120 mg, 0.24 mmol), potassium carbonate (130 mg, 4 equiv.), acetylacetone (132 µL, 4 equiv), were stirred at room temperature in toluene (1 mL) until the starting material was completely consumed (24h). The inorganic salt was filtered off and the mixture was directly purified by flash chromatography on silica gel (Hexane/Ethyl acetate from 9/1 to 8/2) recovering **2** as a pale oil. Yield: 79% (82 mg). ^{1}HNMR ($CDCl_3$, 400 MHz) δ 8.03 (d, *J*= 7.4 Hz, 1H), 7.56-7.22 (m, 8H), 6.68 (d, *J*= 10.5 Hz, 1H), 6.23 (dd, J_1= 10.5, J_2= 4.7 Hz, 1H), 5.42 (d, *J*= 7.4 Hz, 1H), 5.32 (d, *J*= 7.4 Hz, 1H), 4.54 (d, *J*= 4.4 Hz, 1H), 2.38 (s, 3H), 1.93 (s, 3H), 1.38 (s, 9H). ^{13}CNMR ($CDCl_3$, 75 MHz) δ 206.5, 204.3, 166.8, 155.3, 142.9, 135.6, 132.8, 131.3, 128.9, 128.7, 128.5, 128.4, 128.3, 127.5, 127.2, 79.8, 68.4, 67.1, 51.5, 32.3, 28.2. IR (neat): 3120, 3070, 1714,1652 cm^{-1}. HRMS (MALDI): *m/z* calcd for $[C_{25}H_{29}NO_6 + K]^+$: 478.16265; found: 478.16585.

Step 2.

***tert*-butyl 1-(2,4-dioxopentan-3-yl)-3-oxoisoindoline-2-carboxylate 3.** From open intermediate no reaction occurs warming the mixture to 50° C for 2 days (DCE/DCM 0.1 M), using only potassium carbonate (2 equiv.). Addition of racemic Takemoto catalyst (20 mol%) let us to recover the product in almost quantitative yield.

Synthesis of racemic pyrazole derivatives 5 and 6

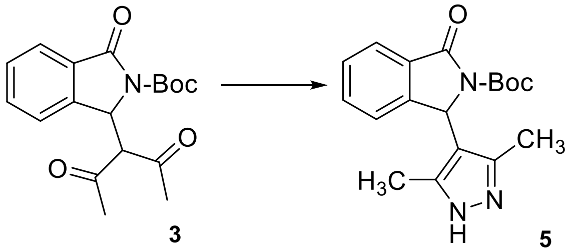

3 **5**

tert-butyl 1-(3,5-dimethyl-1H-pyrazol-4-yl)-3-oxoisoindoline-2-carboxylate 5. To a solution of compound **3** (100 mg, 0.3 mmol, 1 equiv.) in THF (1 mL) hydrazine hydrate 80% (12 µL, 1.2 equiv.) was added and the mixture was stirred for 1h. The solvent was removed under reduced pressure and the crude purified by flash chromatography on silica gel (Hexane/Ethyl acetate from 6/4 to 3/7) recovering as an amorfous solid. Yield: 97% (96 mg). ^{1}HNMR ($CDCl_3$, 300 MHz) δ 7.93 (d, *J*= 7.5 Hz, 1H), 7.59 (t, *J*= 7.5 Hz, 1H), 7.49 (t, *J*= 7.5 Hz, 1H), 7.17 (d, *J*= 7.5 Hz, 1H), 5.98 (s, 1H), 5.13 (br s, 1H), 1.45 (s, 15H). ^{13}CNMR ($CDCl_3$, 100 MHz) δ 166.9, 150.1, 145.1, 143.0, 134.1, 130.4, 128.9, 124.7, 123.4, 112.3, 83.1, 55.9, 28.1, 11.1. IR (KBr disc): 3200, 2940, 2880, 1670, 1656 cm^{-1}. HRMS (MALDI): *m/z* calcd for $[C_{18}H_{21}N_3O_3 + H]^+$: 328.16557; found: 328.16644.

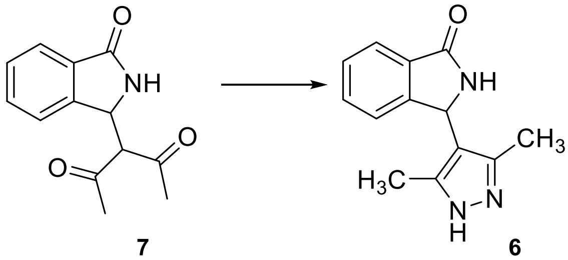

7 **6**

(3,5-dimethyl-1H-pyrazol-4-yl)isoindolin-1-one 6. Prepared following the same procedure reported for **5** starting from 100 mg (0.44 mmol) of compound **7**, prepared as described by Massa et al.[2] Purification by flash chromatography on silica gel (Ethyl acetate/MeOH 9/1) let to recover a white solid. Yield: 94% (45 mg). M.p. >240 °C. ^{1}HNMR (MeOD, 400 MHz) δ 7.83 (d, *J*= 7.4 Hz, 1H), 7.61 (t, *J*= 7.4 Hz, 1H), 7.53 (t, *J*= 7.4 Hz, 1H), 7.31 (d, *J*= 7.04 Hz, 1H), 5.76 (s, 1H), 1.96 (s, 6H). ^{13}CNMR (MeOD, 100 MHz) δ 171.4, 147.8, 132.2, 131.7, 127.9, 123.1, 122.7, 110.6, 52.1, 9.1. IR (KBr disc): 3200, 2940, 2880, 1656 cm^{-1}. HRMS (MALDI): *m/z* calcd for $[C_{13}H_{13}NO + H]^+$: 228.11314; found: 228.11186.

One-pot procedure for the synthesis of compound 3

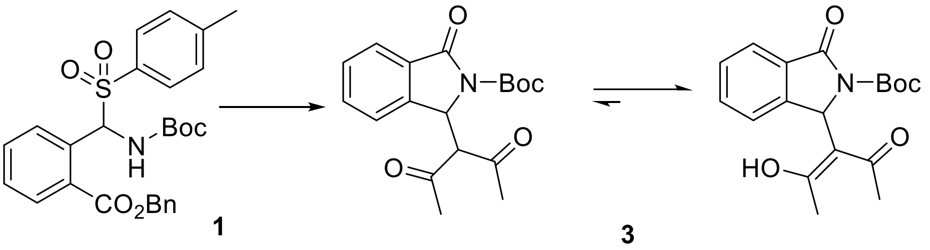

***tert*-butyl 1-(2,4-dioxopentan-3-yl)-3-oxoisoindoline-2-carboxylate 3.** In an ACE tube, **1** (100 mg, 0.2 mmol, equiv.), K_2CO_3 (140 mg, 5 equiv), acetylacetone (132 µL, 5 equiv), (*R, R*) -Takemoto catalyst (20 mol%) were stirred at -40 °C in dichoroethane (1.2 mL)/ dichloromethane (800 µL) until the starting material was completely consumed (24 h). Then, the reaction mixture was warmed to 50-60°C for 2 days. The inorganic salt was filtered off and the mixture was directly purified by flash chromatography on silica gel (Hexane/Ethyl acetate from 9/1 to 7/3) recovering **3** as an amorphous solid. Yield: 94% (62 mg). Spectroscopic data were found in agreement with racemate. HRMS (MALDI): *m/z* calcd for $[C_{18}H_{21}NO_5 + K]^+$: 370.10513; found: 370.10561. Enantiomeric excess was determinedon chiral HPLC**.** Chiral column OD Hexane/Isopropanol 80/20 0.6 mL/min, λ 220 and 254 nm. Racemization occurs during the cyclization.

Two steps procedures for enantioselective synthesis of compound 3 and pyrazole 5

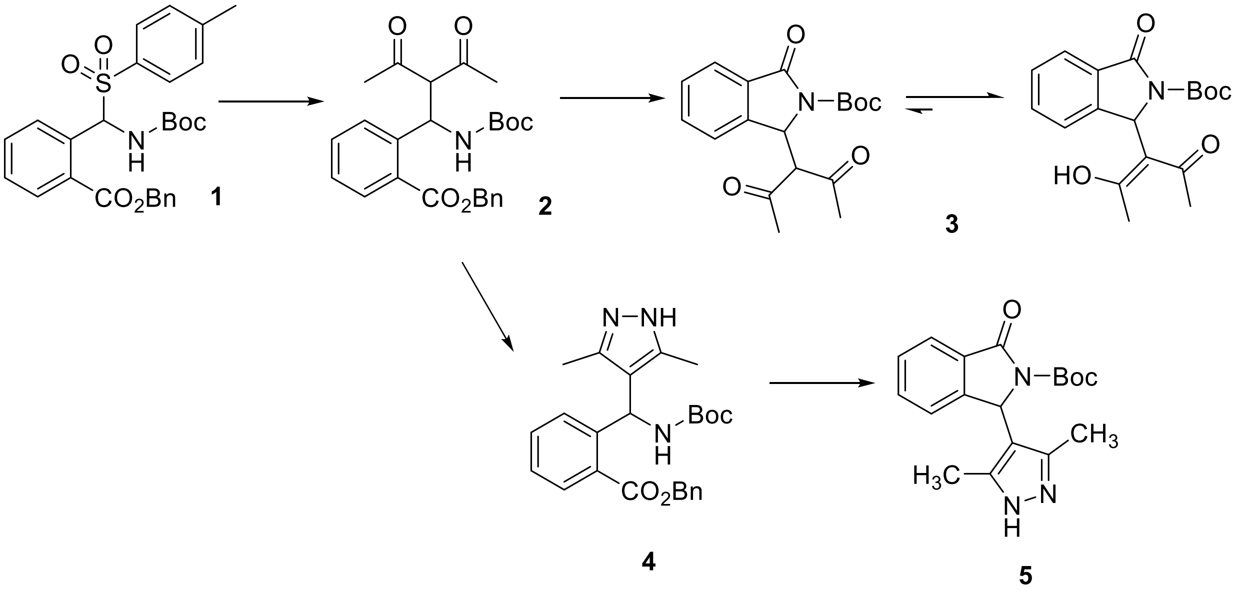

Compound 3.

Step 1.

Benzyl 2-(2-acetyl-1-((tert-butoxycarbonyl)amino)-3-oxobutyl)benzoate 2. In an ACE tube, **1** (120 mg, 0.24 mmol, equiv.), K_2CO_3 (66 mg, 2 equiv), acetylacetone (66 µL, 2 equiv), (*R, R*)-Takemoto catalyst (20 mol%) were stirred at -40°C in dichoroethane (1.2 mL)/dichloromethane (800 µL) until the starting material was completely consumed (24 h). The inorganic salt was filtered off and the mixture was directly purified by flash chromatography on silica gel (Hexane/Ethyl acetate from 9/1 to 8/2) recovering a pale oil. Yield: 80% (84 mg). Spectroscopic data were found in agreement with racemate. HRMS (MALDI): *m/z* calcd for $[C_{25}H_{29}NO_6 + K]^+$: 478.16265; found: 478.16545. $[\alpha]_D^{20}$: +80.9 (c 1, $CHCl_3$). Enantiomeric excess was determined on chiral HPLC Chiral column IE-3, Hexane/Isopropanol, 80/20 0.6 mL/min, λ 220 and 254 nm. Ee: 89%, t_1: 24.3 min and t_2: 27.4 min.

Step 2.

***tert*-butyl 1-(2,4-dioxopentan-3-yl)-3-oxoisoindoline-2-carboxylate 3.** To a solution of compound **2** (0.1 mmol) in toluene (0.1 M), K_2CO_3 (0.2 equiv) and (*R*), (*R*)-Takemoto catalyst was warmed to 40° C for 2 days. The inorganic salt was filtered off and the mixture was directly purified by flash chromatography on silica gel (Hexane/Ethyl acetate from 8/2 to 7/3) recovering **3**. Yield: 94% (31 mg). Spectroscopic data were found in agreement with racemate. Enantiomeric excess was determined on chiral HPLC Chiral column OD Hexane/Isopropanol 80/20 0.6 mL/min, λ 220 and 254 nm. Racemization occurs during the cyclization.

Pyrazole 5

Step 1.

Benzyl 2-(((tert-butoxycarbonyl)amino)(3,5-dimethyl-1H-pyrazol-4-yl)methyl)benzoate 4. To a solution of intermediate **2** (40 mg, 0.12 mmol, 1 equiv.) in THF (600 µL) hydrazine hydrate 80% was added (8 µL, 1.1 equiv.) and the mixture was stirred for 1h. The solvent was removed under reduced pressure and the crude purified by flash chromatography on silica gel (Hexane/Ethyl acetate from 6/4 to 3/7) recovering a pale oil. Yield: 92% (48 mg). ^{1}HNMR ($CDCl_3$, 400 MHz) δ 7.78 (d, *J*= 7.7 Hz, 1H), 7.59 (app dd, J_1= 7.7 Hz, J_2= 1.3 Hz, 1H), 7.49 (dt, *J*= 6.5 Hz, 1H), 7.41-7.23 (m, 7H), 6.63 (d, *J*= 7.7 Hz, 1H), 6.16 (br s, 1H), 5.10 (s, 2H), 1.87 (s, 6H), 1.44 (s, 9H). ^{13}CNMR ($CDCl_3$, 100 MHz) δ 167.4, 155.0, 142.9, 142.4, 135.9, 131.6, 130.8, 130.1, 128.7, 128.2, 128.1, 127.4, 127.2, 115.8, 79.8, 66.8, 48.4, 28.5, 11.5. IR (neat): 3200, 2940, 2880, 1689, 1672,1656 cm^{-1}. HRMS (MALDI): *m/z* calcd for $[C_{25}H_{29}N_3O_4 + K]^+$: 474.17896; found: 474.17335. $[\alpha]_D^{20}$: +12.6 (c 0.6, $CHCl_3$). Enantiomeric excess was determined after cyclization.

Step 2.

tert-butyl 1-(3,5-dimethyl-1H-pyrazol-4-yl)-3-oxoisoindoline-2-carboxylate 5. A solution of compound **4** (50 mg, 0.11 mmol, 1 equiv.) in toluene (1.1 mL), potassium carbonate (4 mg, 20 mol%) and (*R*,*R*)-Takemoto's catalyst (20 mol%) was warmed to 40°C for 48h, till starting material was consumed. The crude was purified by flash chromatography on silica gel (Hexane/Ethyl acetate from 6/4 to 3/7) recovering an amorphous solid. Yield: 97% (35 mg). Spectroscopic data were found in agreement with racemate. HRMS (MALDI): *m/z* calcd for $[C_{18}H_{21}N_3O_3 + H]^+$: 328.16557; found: 328.16678. $[\alpha]_D^{20}$: +35.7 (c 1, $CHCl_3$). Enantiomeric excess was determined by chiral HPLC. Chiral column OD, Hexane/Isopropanol, 80/20 0.6 mL/min, λ 220 and 254 nm. Ee: 89%, t_1: 9.1 min and t_2: 10.9 min.

Deprotection of isoindolinones 5

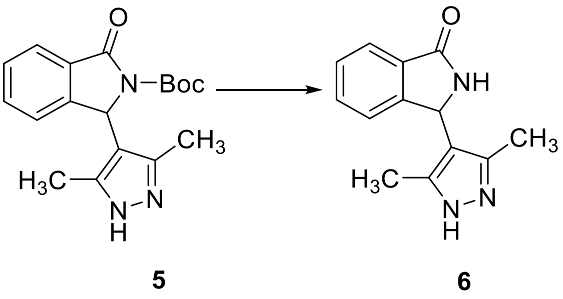

3-(3,5-dimethyl-1H-pyrazol-4-yl)isoindolin-1-one 6. At 0 °C, to a chilled solution of compound **5** (30 mg, 0.09 mmol, 1 equiv.) in dichoromethane (800 µL) was added trifluoroacetic acid (TFA, 130 µL) and the mixture was stirred for 3h. The suspension was diluted with dichoromethane, basified with NaOH 1N and washed with brine. The crude obtained after evaporation of the solvent, was purified by flash chromatography on silica gel (Ethyl acetate/Mehanol 95/5) recovering an amorphous solid. Yield: 98% (11 mg). Spectroscopic data were found in agreement with racemate. HRMS (MALDI): *m/z* calcd for $[C_{13}H_{13}NO + H]^+$: 228.11314; found: 228.11156. $[\alpha]_D^{20}$: +19.4 (c 0.3, $CHCl_3$). Enantiomeric excess was determined by chiral HPLC. Chiral column OD Hexane/Isopropanol 60/40 0.6 mL/min, λ 220 and 254 nm. Ee: 89%, t_1: 5.4 min and t_2: 7.6 min.

Reaction with dibenzylamine (DBA)

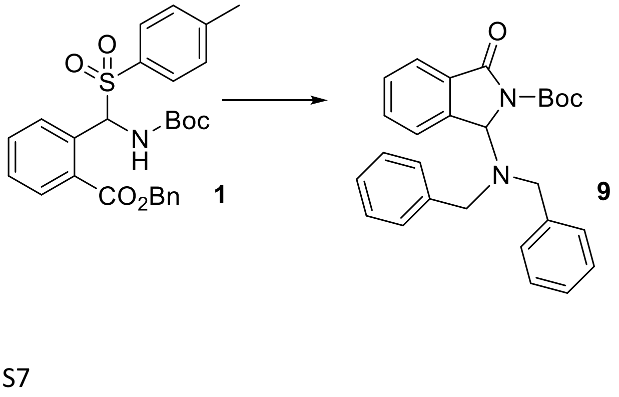

tert-butyl 1-(dibenzylamino)-3-oxoisoindoline-2-carboxylate 9. To a solution of α-amido sulfone **1** (40 mg, 0.08 mmol, 1.0 equiv.) in toluene (1.6 mL), dibenzylamine (1.5 equiv., 0.12 mmol), K_2CO_3 (55 mg, 0.4 mmol, 5 equiv.) and (*S*,*S*) -Takemoto's catalyst (20 mol%) were added and the reaction mixture was stirred at -20 °C, till the starting material completely disappear. Then the reaction was allowed to stir at room temperature, till the intermediate was full converted in the cyclic product. Purification on silica gel (Hexane/ Ethyl acetate, 5:1) afforded the resulting cyclic product as an oil. Yield: 94% (32 mg.).^{1}H NMR (300 MHz, $CDCl_3$) δ 7.78 (d, *J*= 7.28 Hz, 1H), 7.60-7.55 (m, 1H), 7.47-7.20 (m, 12H), 6.13 (s, 1H), 3.84 (d, *J*= 12.5 Hz, 2H), 3.66 (d, *J*= 12.5 Hz, 2H), 1.65 (s, 9H). ^{13}CNMR (100 MHz, $CDCl_3$) δ 166.3, 151.9, 143.6, 139.2, 133.9, 131.2, 129.7, 129.3, 129.2, 128.7, 128.6, 128.5, 128.4, 127.3, 124.4, 124.2, 83.3, 76.4, 53.2, 28.3. HRMS (MALDI): *m/z* calcd for $[C_{27}H_{28}N_2O_3 + H]^+$: 429.2173; found: 429.2190. $[\alpha]_D^{18}$: = + 88 (c = 0.8, $CHCl_3$). Enantiomeric excess was determined by chiral HPLC. Chiral column OD-H, Hexane/Isopropanol, 95:5, 0.6 mL/min, λ 220 and 254 nm. Ee: 63%, t_1: 9.1 min and t_2: 10.0 min).

Table S1

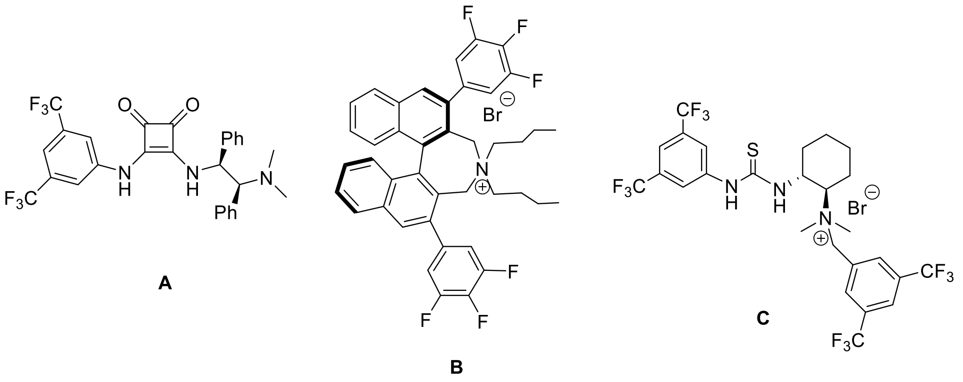

Entry	Cat (20 mol%)	Solvent	Time(h) Step 1/step 2	T(C°) step 1/step 2	9, Yield (%)[1]	9, ee (%)[2]
1[3]	**A**	Toluene	7/137	-20/RT	50	0
2[3]	**B**	Toluene	7/112	-20/RT	82	-6
3[3]	**C**	Toluene	7/21	-20/RT	95	0

[1] Isolated yield. [2] Determined by HPLC on chiral column.

Crystallographic data

Crystals of the compound (+)-**6** suitable for single crystal X-ray diffraction analysis were obtained by slow evaporation, dissolving 4 mg of the compound in 0.6 mL of methanol and then adding dichloromethane in a final ratio of 3:1. A colourless needle-like single crystal with dimensions of 0.45 mm × 0.10 mm × 0.05 mm was selected and mounted on a cryoloop with paratone oil and measured at 100 K with a Bruker D8 QUEST diffractometer equipped with a PHOTON detector using Cu-$K\alpha$ radiation (λ= 1.54178 Å). Data indexing was performed using APEX3.[3] Data integration and reduction were performed using SAINT.[3] Absorption correction was performed by multi-scan method in SADABS.[3] The structures were solved using SHELXS[4] and refined by means of full matrix least-squares based on F^2 using the program SHELXL.[5]

Non-hydrogen atoms were refined anisotropically. Hydrogen atoms attached to the nitrogen atoms N1 and N2 were localized from the difference density map and refined, whereas the remaining hydrogen atoms were positioned geometrically and included in structure factors calculations but not refined. ORTEP diagrams (Figure S1) were drawn using OLEX2.[6] Relevant crystallographic data are reported in Table S2.

The chirality of carbon atoms C2 (R) was assigned due to anomalous dispersion effects in diffraction measurements on the crystals. Flack parameters[7] is reported in Table S2.

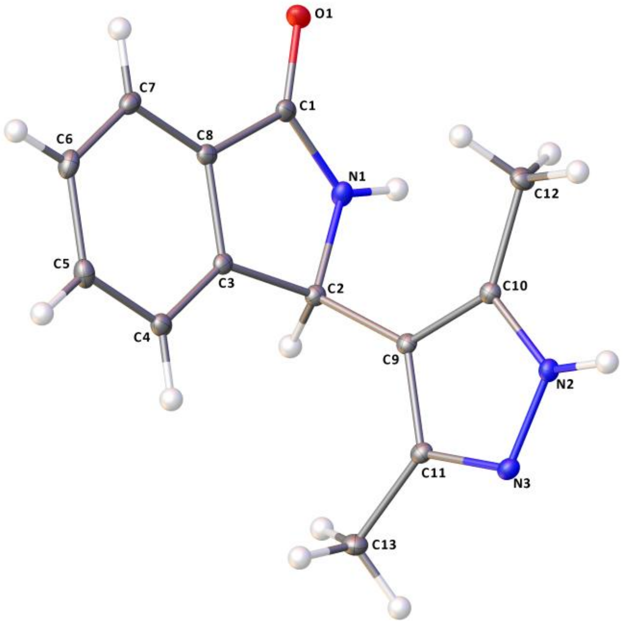

Figure S1 ORTEP drawings for compound (+)-**6**. Atom types: C grey, O red, N blue and H white. Ellipsoids are drawn at 20% probability level (CCDC 2246867).

Table S2 Crystallographic data for compound (+)-**6**.

	(+)-**6**
**T (K)**	100
**Formula**	$C_{13}H_{13}N_3O$
**Formula weight**	227.26
**System**	Orthorhombic
**Space group**	$P\ 2_12_12_1$
a **(Å)**	5.4607(7)
b **(Å)**	12.354(4
c **(Å)**	16.924(3)
α **(°)**	90
β **(°)**	90
γ **(°)**	90
V **(Å^3)**	1141.7(4)
**Z**	4
Dx **(g cm^{-3})**	1.322
**λ (Å)**	1.54178
μ **(mm^{-1})**	0.701
F_{000}	480.0
**R1 (I > 2σI)**	0.0283(2104)
$_wR_2$	0.0774(2127)
**N. of param.**	165
**GooF**	1.084
ρ_{min}, ρ_{max} **(eÅ^{-3})**	-0.16, 0.19
**Flack parameter**	0.09(6)

References

1. Serusi, Lorenzo; Palombi, Laura; Pierri, Giovanni; Di Mola, Antonia; Massa, Antonio.” *Journal of Organic Chemistry*, **2022**, 87. 8420-8428.
2. V. More, A. Di Mola, M. Perillo, P. De Caprariis, R. Filosa, A. Peduto and A. Massa. *Synthesis*, **2011**, 3027.
3. Bruker (**2015**). APEX3, SAINT and SADABS. Bruker AXS Inc, Madison, Wisconsin, USA.
4. Sheldrick, G. M. *Acta Cryst. A64*, **2008**, 112-122.
5. Sheldrick, G. M. *Acta Cryst.* C71, **2015**, 3-8.
6. Dolomanov, O. V.; Bourhis, L. J.; Gildea, R. J.; Howard, J. A. K; Puschmann, H. *J. Appl. Cryst.*, **2009**, *42*, 339-341.
7. Flack, H. D. *Acta Cryst. A39*, **1983**, 876-881.

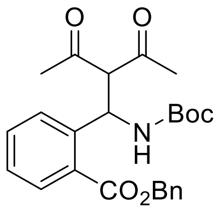
O
O
N
Boc
H
CO2Bn

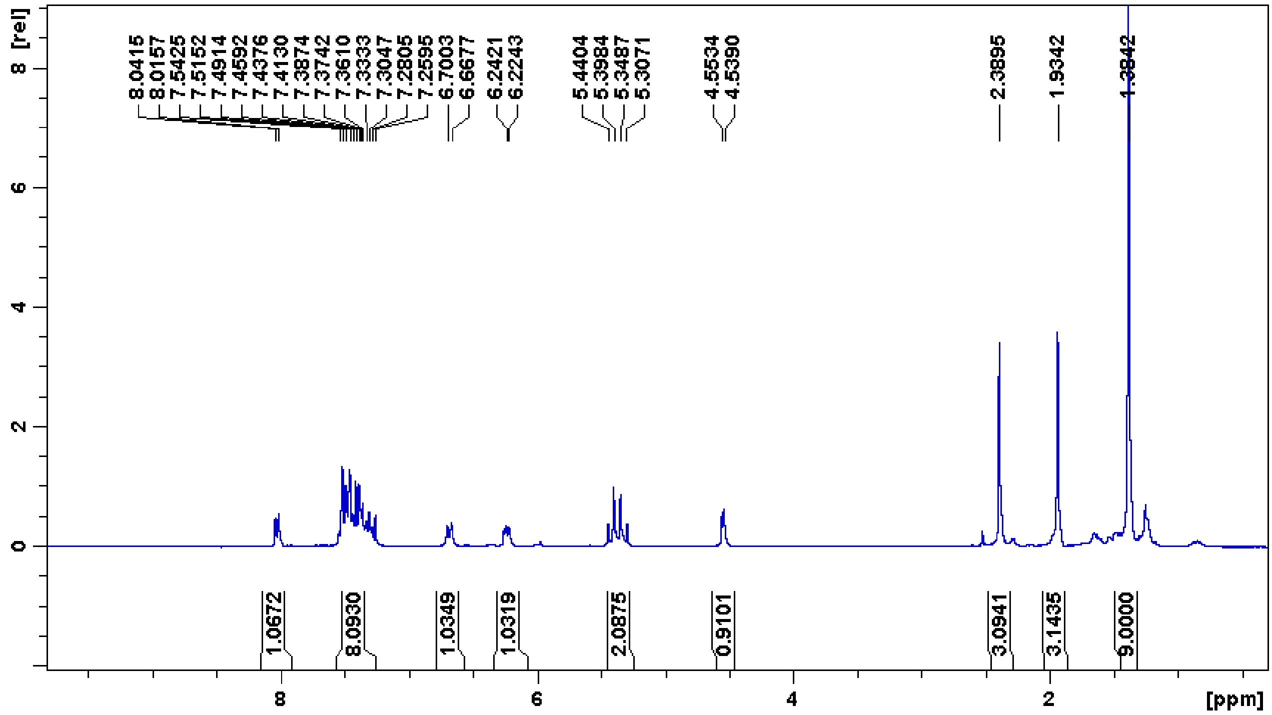

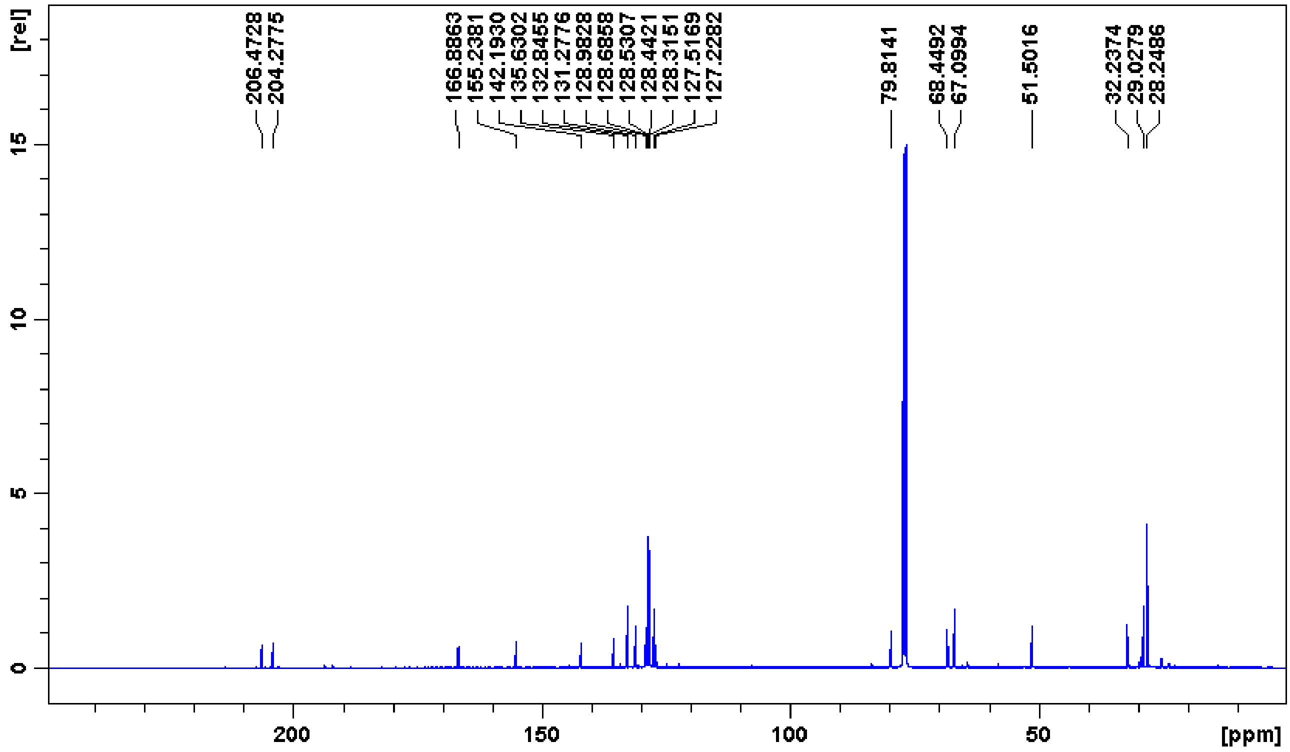

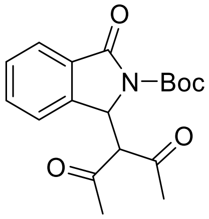
O
N−Boc
O
O

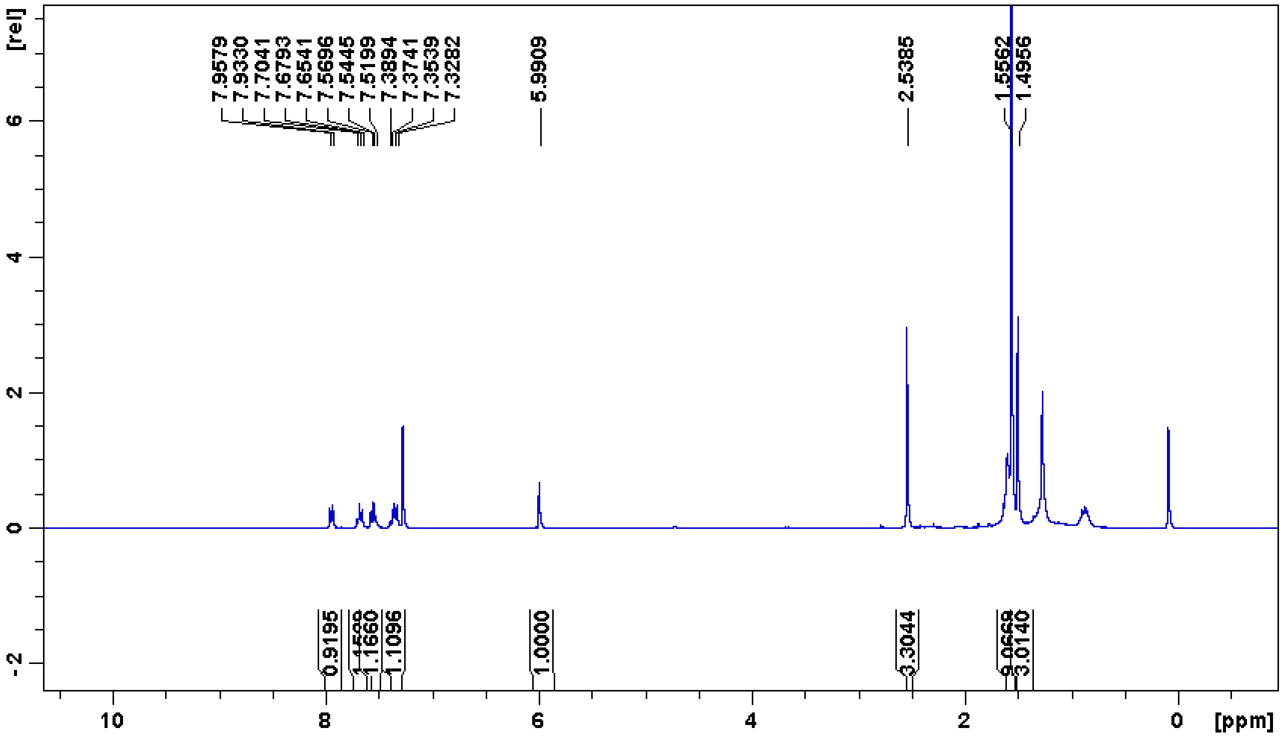
[rel]
7.9579
7.9330
7.7041
7.6793
7.6541
7.5696
7.5445
7.5199
7.3894
7.3741
7.3539
7.3282
5.9909
2.5385
1.5562
1.4956
0.9195
1.1660
1.1096
1.0000
3.3044
3.0140
[ppm]

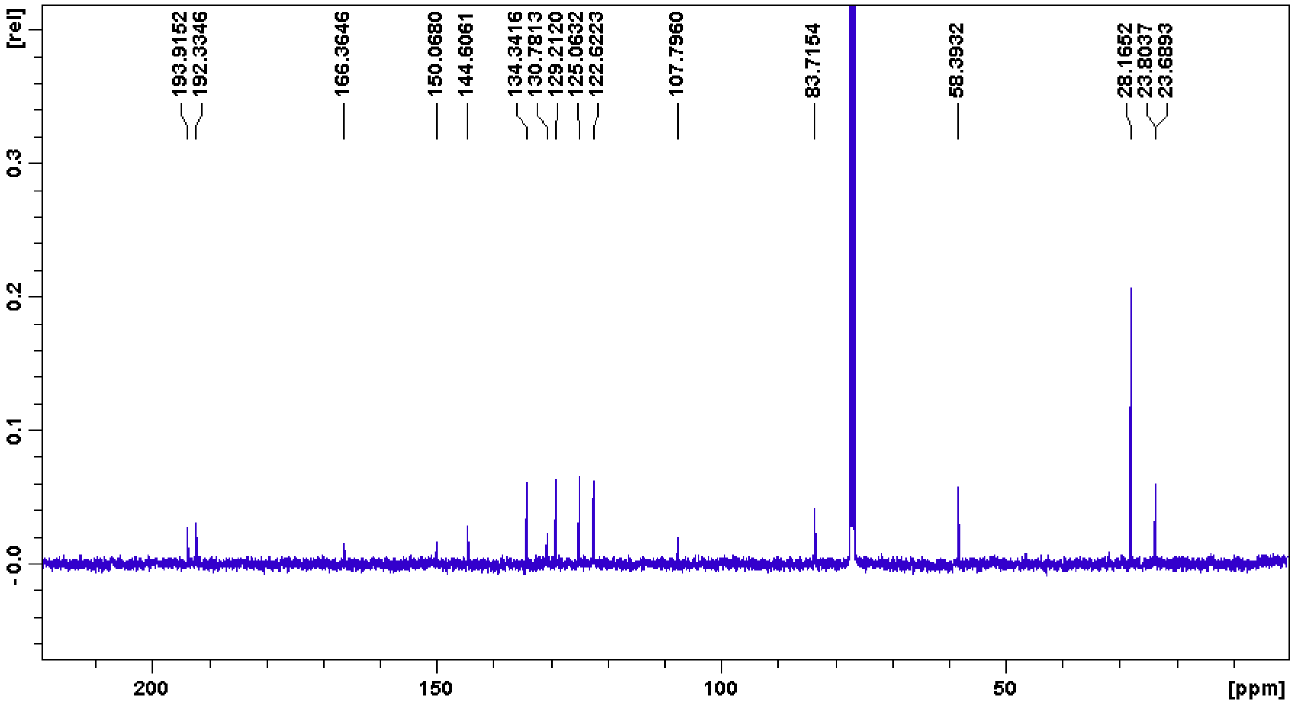
[rel]
193.9152
192.3346
166.3646
150.0680
144.6061
134.3416
130.7813
129.2120
125.0632
122.6223
107.7960
83.7154
58.3932
28.1652
23.8037
23.6893
[ppm]

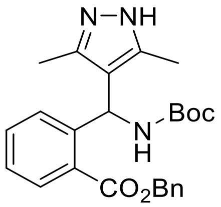
N–NH
N
H
Boc
CO2Bn

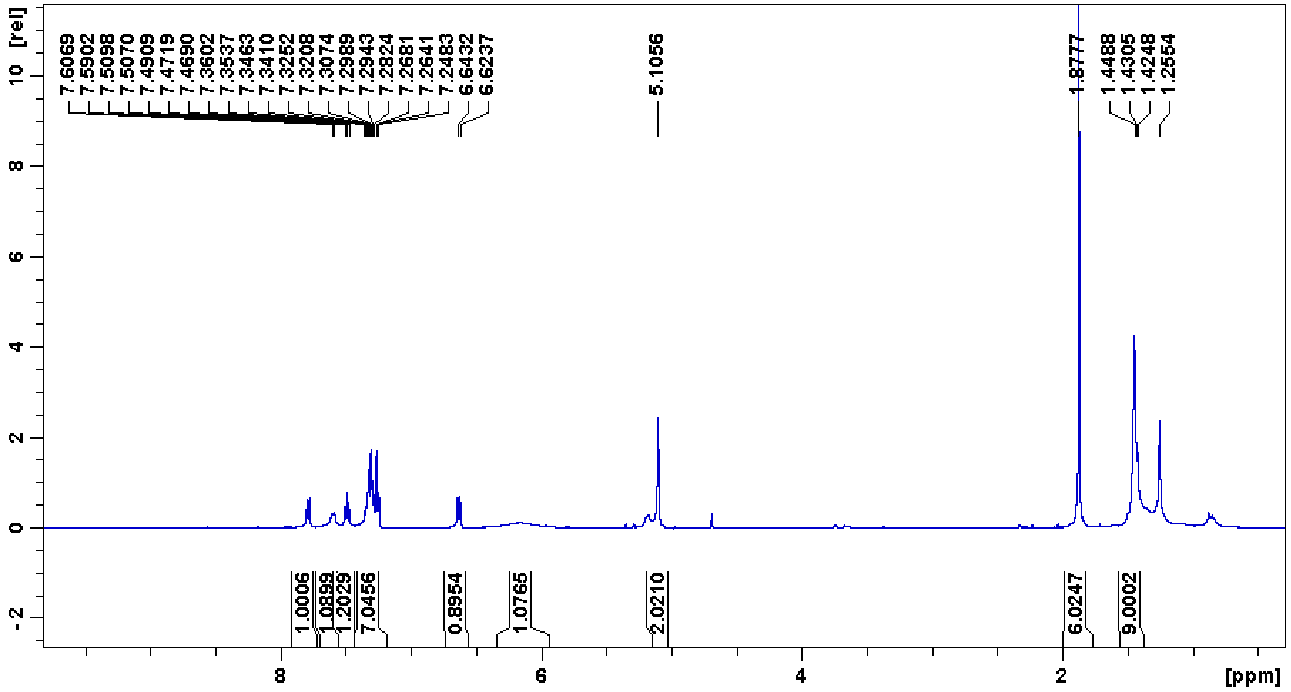
7.6069
7.5902
7.5098
7.5070
7.4909
7.4719
7.4690
7.3602
7.3537
7.3463
7.3410
7.3252
7.3208
7.3074
7.2989
7.2943
7.2824
7.2681
7.2641
7.2483
6.6432
6.6237
5.1056
1.8777
1.4488
1.4305
1.4248
1.2554
1.0006
1.0899
1.2029
7.0456
0.8954
1.0765
2.0210
6.0247
9.0002
[rel]
[ppm]

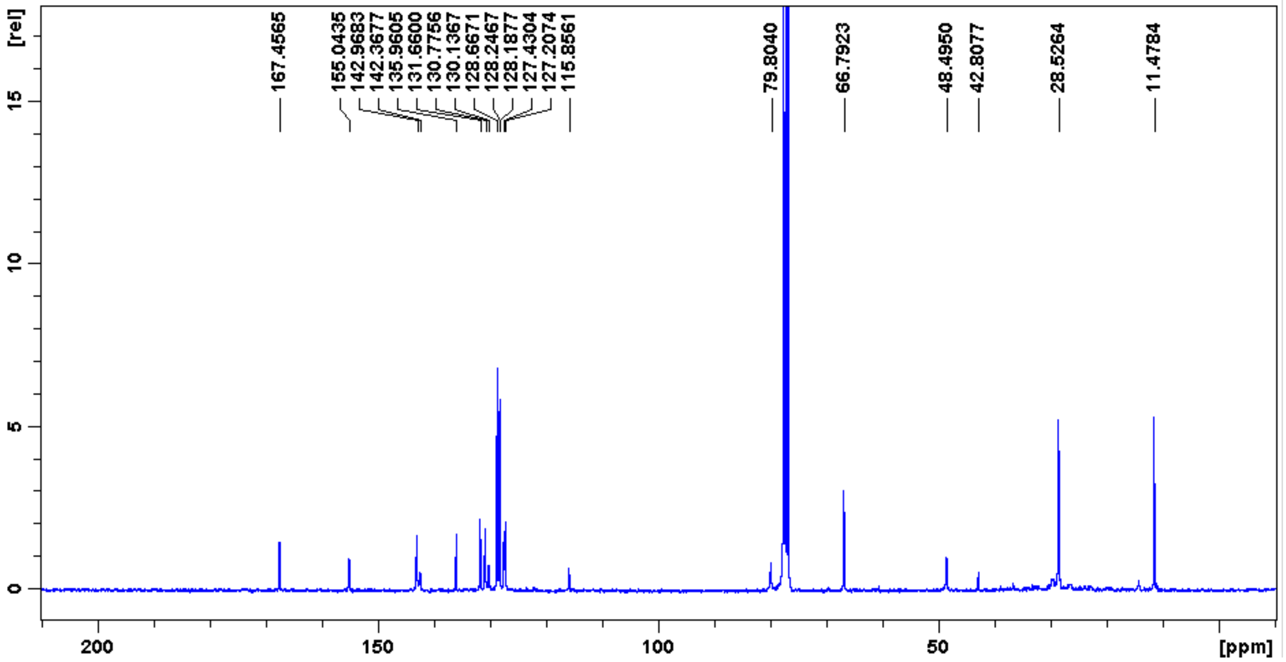
167.4565
155.0435
142.9683
142.3677
135.9605
131.6600
130.7756
130.1367
128.6671
128.2467
128.1877
127.4304
127.2074
115.8561
79.8040
66.7923
48.4950
42.8077
28.5264
11.4784
[rel]
[ppm]

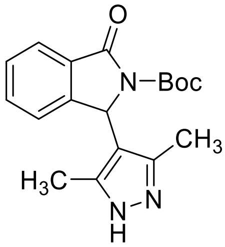
O
N−Boc
CH3
H3C
N
N
H

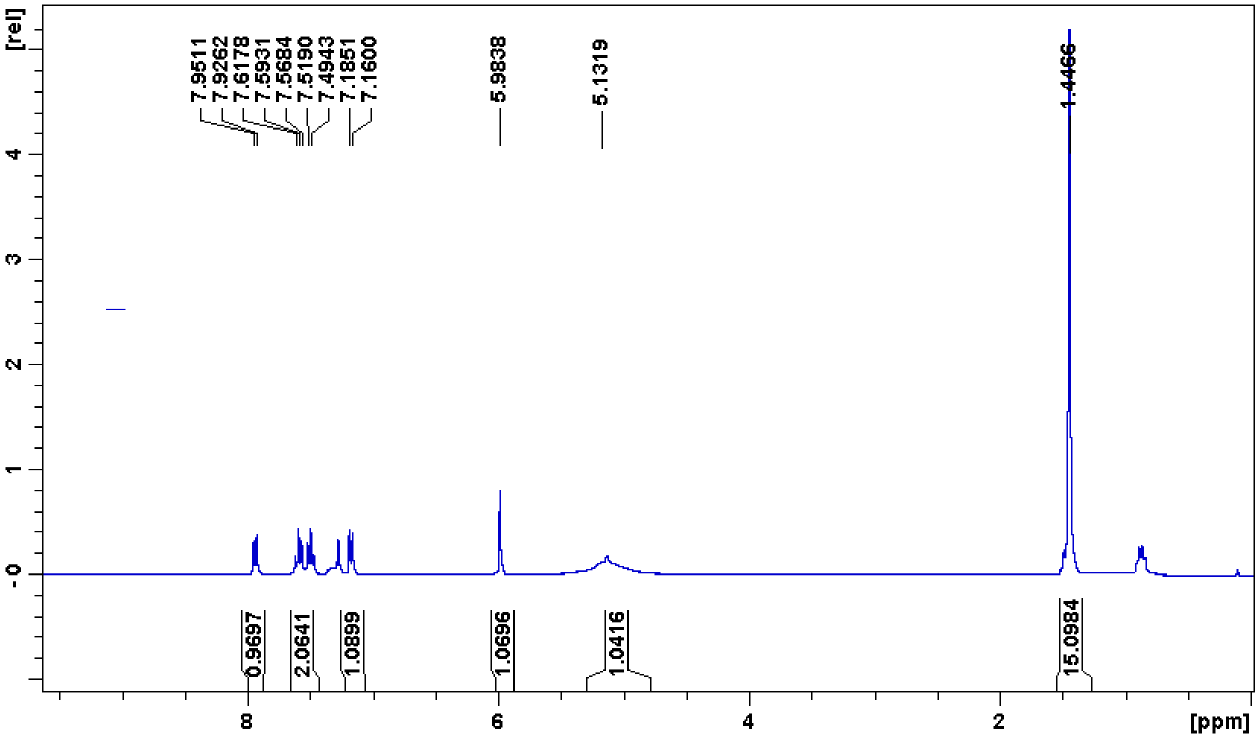
[rel]
7.9511
7.9262
7.6178
7.5931
7.5684
7.5190
7.4943
7.1851
7.1600
5.9838
5.1319
1.4466
0.9697
2.0641
1.0899
1.0696
1.0416
15.0984
8
6
4
2
[ppm]

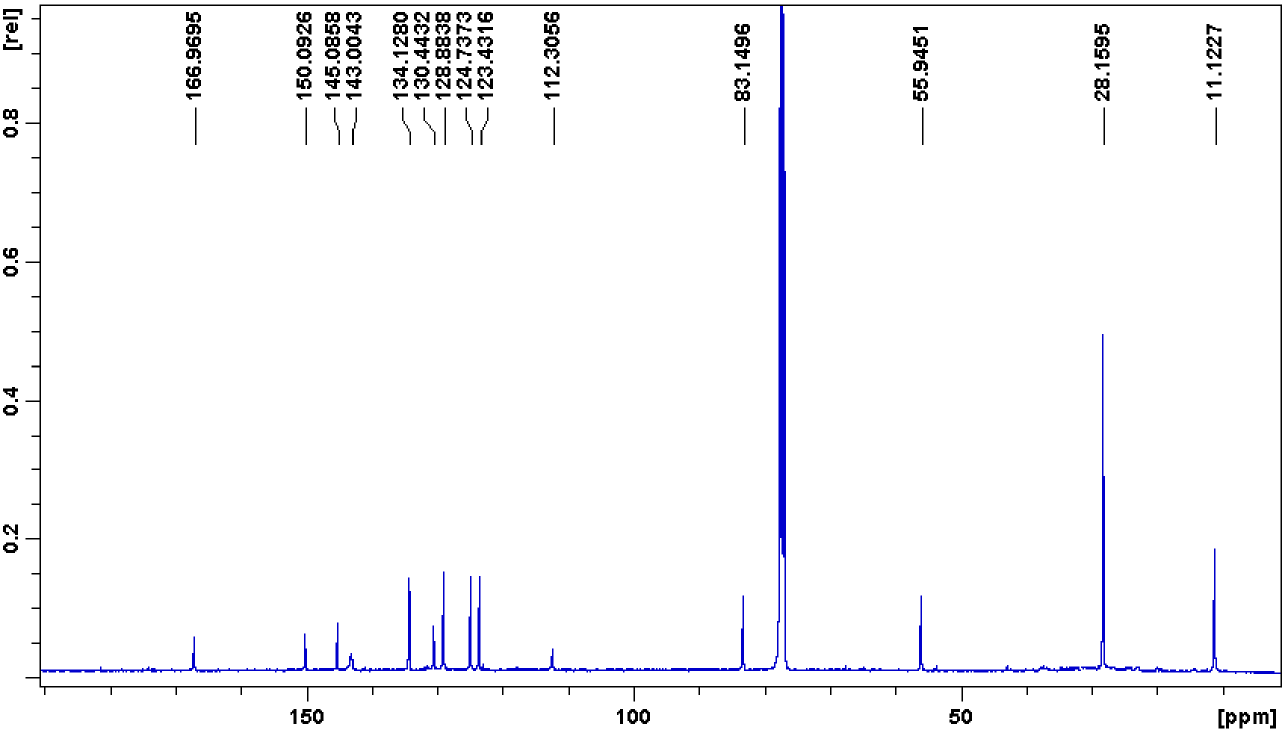
[rel]
166.9695
150.0926
145.0858
143.0043
134.1280
130.4432
128.8838
124.7373
123.4316
112.3056
83.1496
55.9451
28.1595
11.1227
0.8
0.6
0.4
0.2
150
100
50
[ppm]

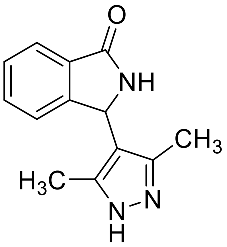
O
NH
CH3
H3C
N
N
H

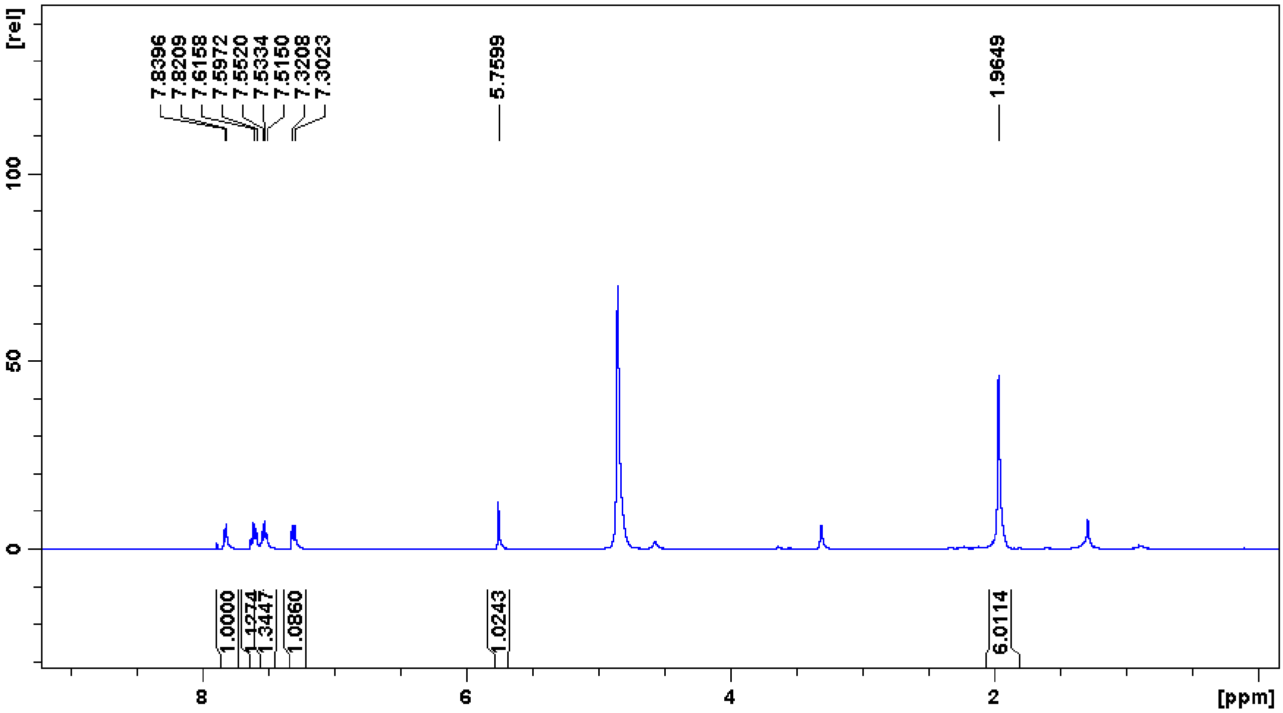
[rel]
7.8396
7.8209
7.6158
7.5972
7.5520
7.5334
7.5150
7.3208
7.3023
5.7599
1.9649
1.0000
1.1274
1.3447
1.0860
1.0243
6.0114
[ppm]

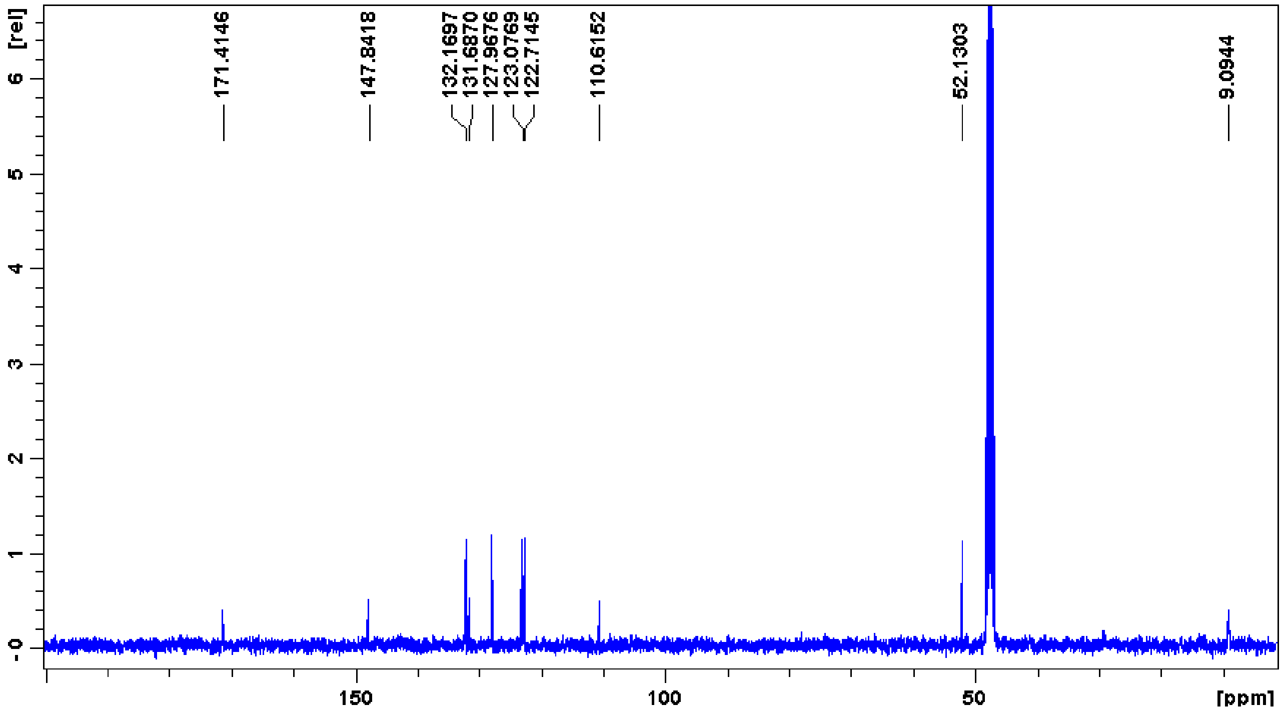
[rel]
171.4146
147.8418
132.1697
131.6870
127.9676
123.0769
122.7145
110.6152
52.1303
9.0944
[ppm]

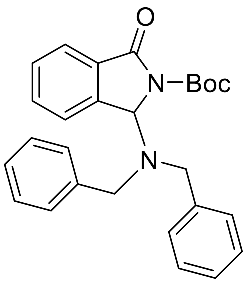
O
N−Boc
N

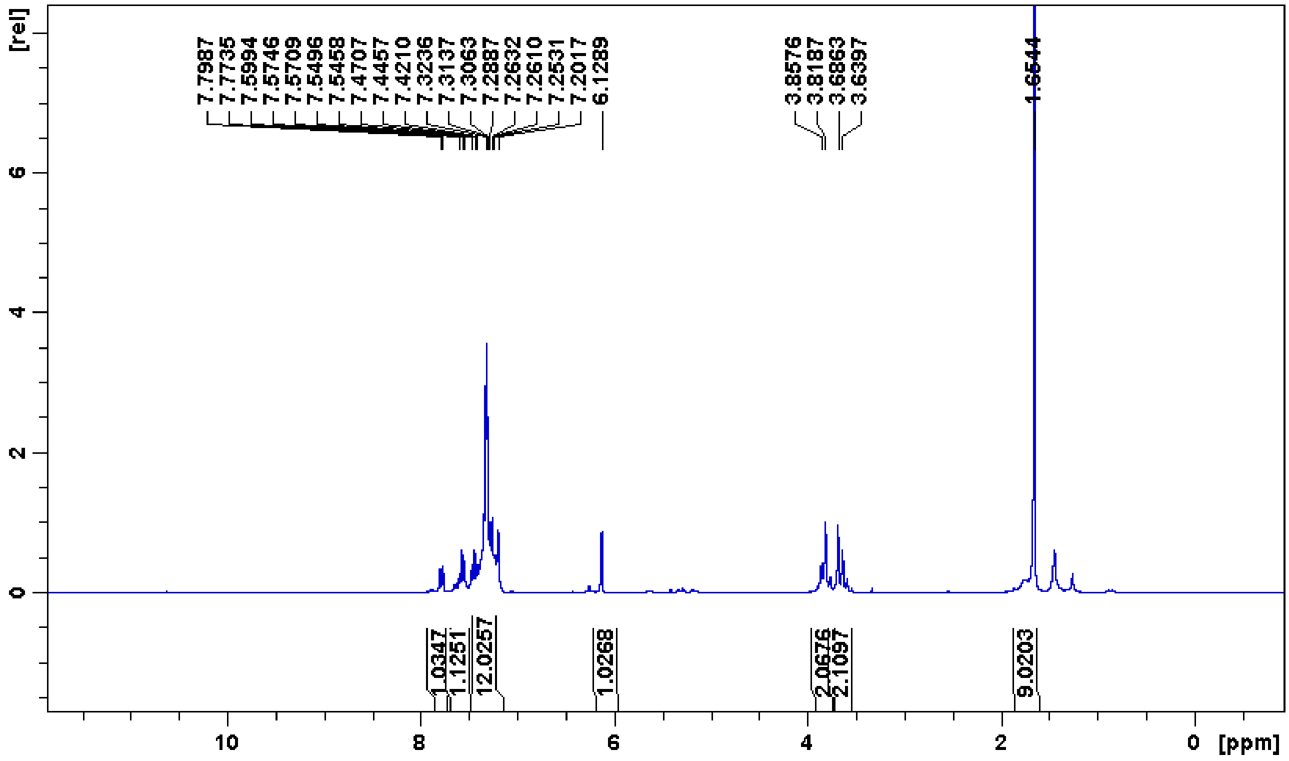
7.7987
7.7735
7.5994
7.5746
7.5709
7.5496
7.5458
7.4707
7.4457
7.4210
7.3236
7.3137
7.3063
7.2887
7.2632
7.2610
7.2531
7.2017
6.1289
3.8576
3.8187
3.6863
3.6397
1.6544
1.0347
1.1251
12.0257
1.0268
2.0676
2.1097
9.0203
[rel]
[ppm]

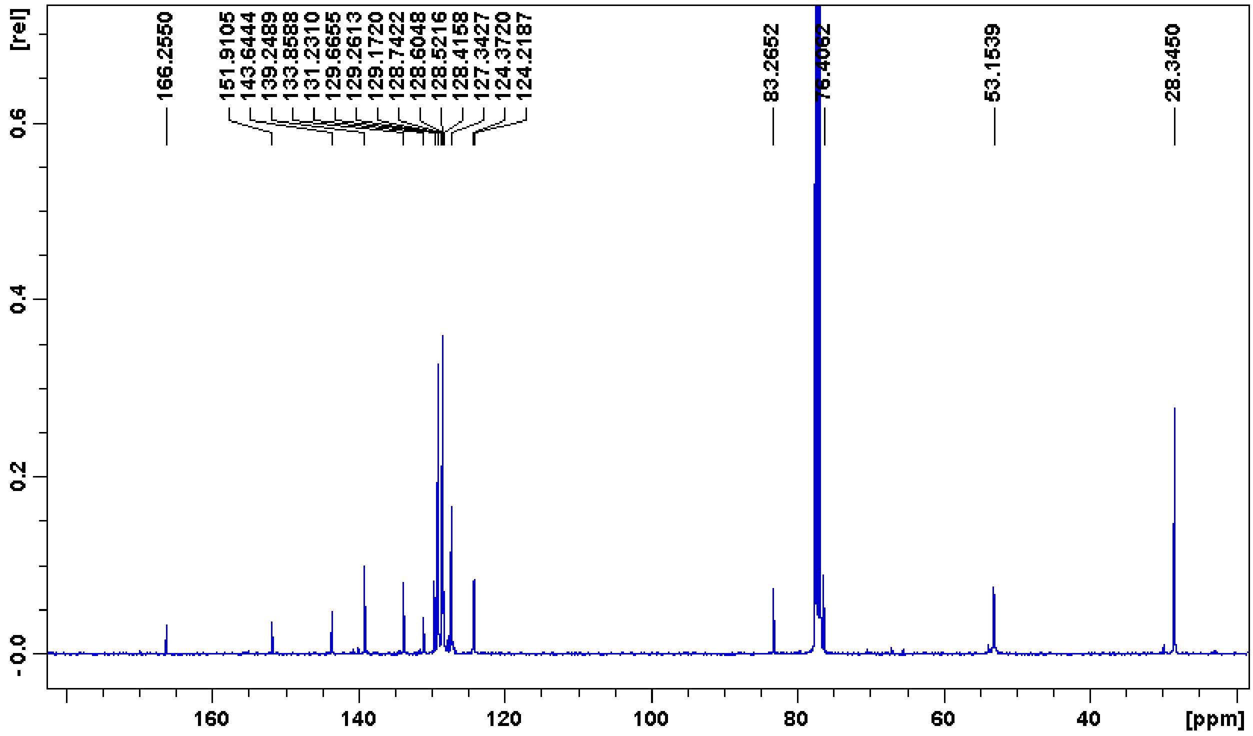
166.2550
151.9105
143.6444
139.2489
133.8588
131.2310
129.6655
129.2613
129.1720
128.7422
128.6048
128.5216
128.4158
127.3427
124.3720
124.2187
83.2652
76.4062
53.1539
28.3450
[rel]
[ppm]

HPLC TRACES

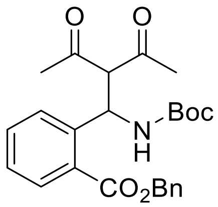

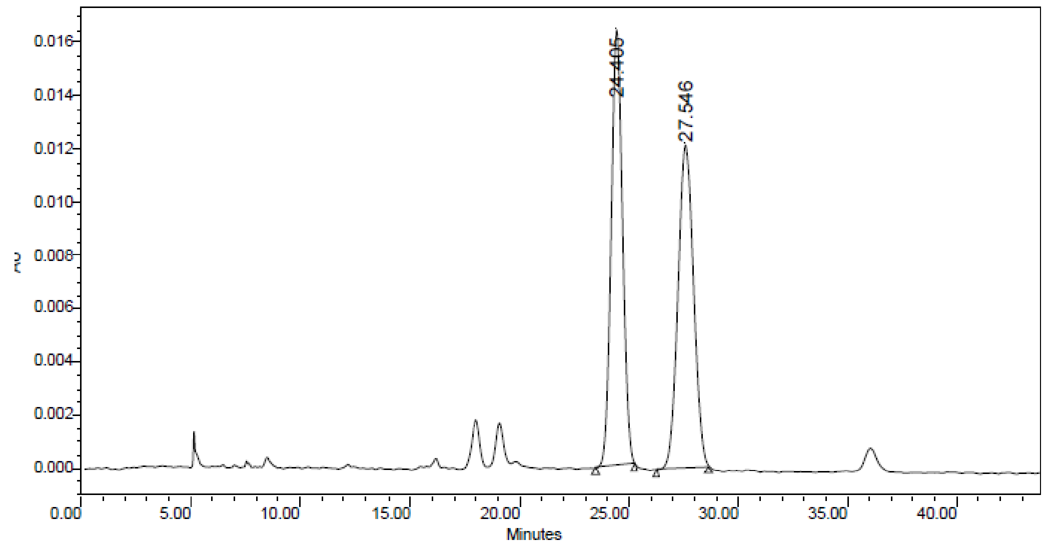

	RT (min)	Area (µV*sec)	% Area	Height (µV)	% Height
1	24.405	608379	49.71	16397	57.42
2	27.546	615459	50.29	12159	42.58

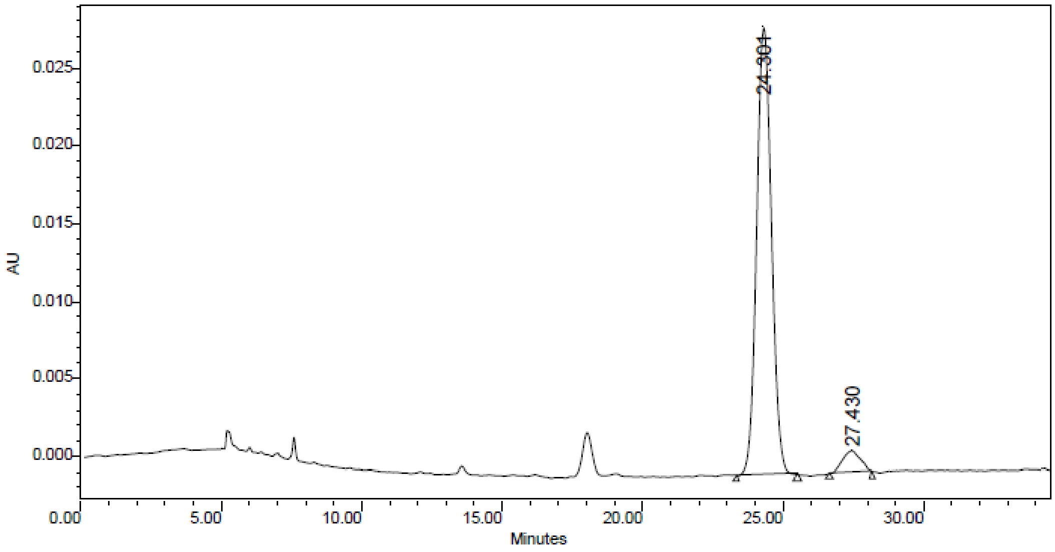

	RT (min)	Area (µV*sec)	% Area	Height (µV)	% Height
1	24.301	1063189	94.50	28743	95.40
2	27.430	61863	5.50	1387	4.60

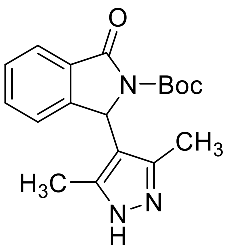

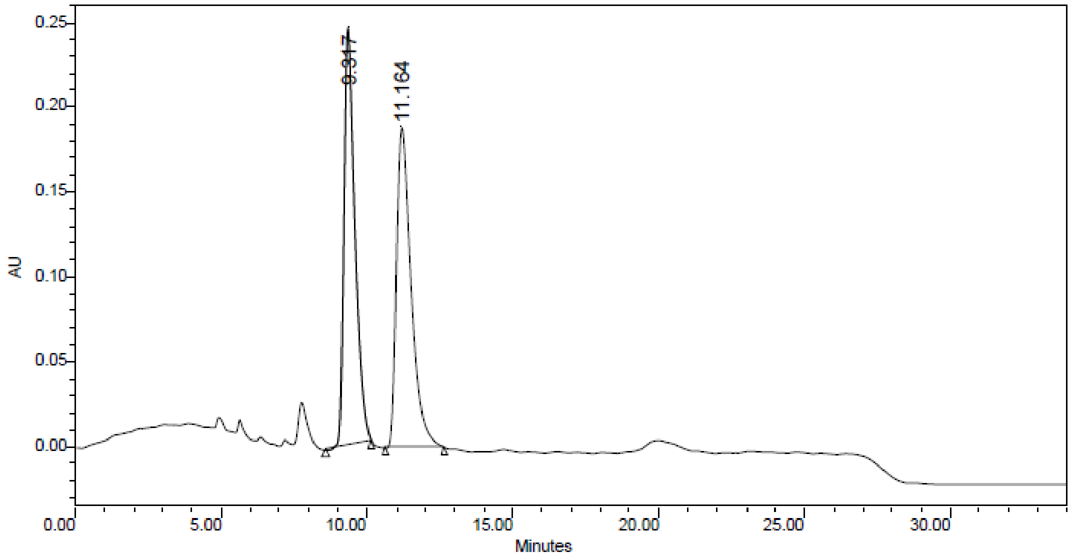

	RT (min)	Area (µV*sec)	% Area	Height (µV)	% Height
1	9.317	6491241	49.51	246005	56.61
2	11.164	6620857	50.49	188587	43.39

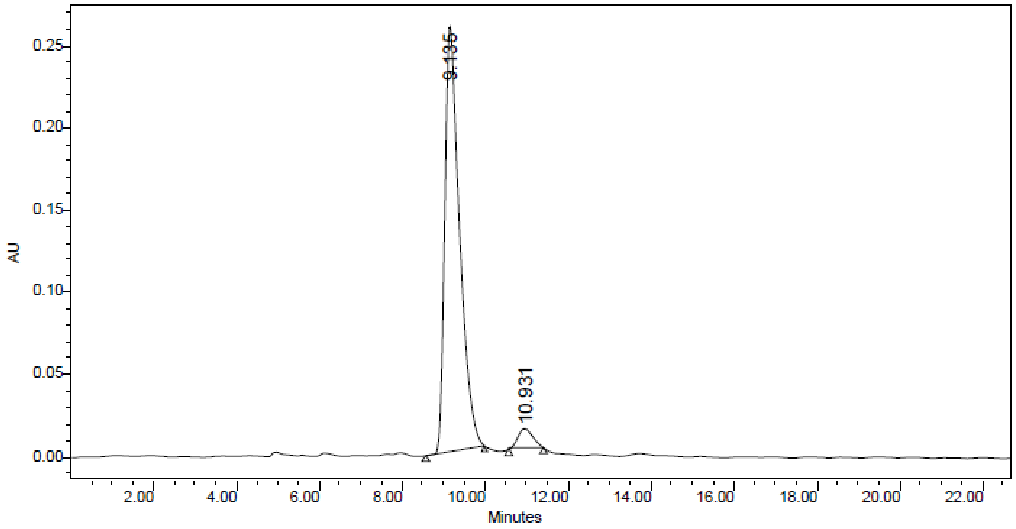

	RT (min)	Area (µV*sec)	% Area	Height (µV)	% Height
1	9.135	6452428	95.41	258465	95.52
2	10.931	310713	4.59	12122	4.48

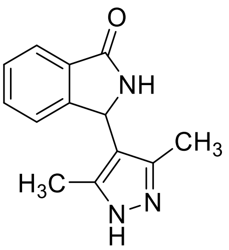

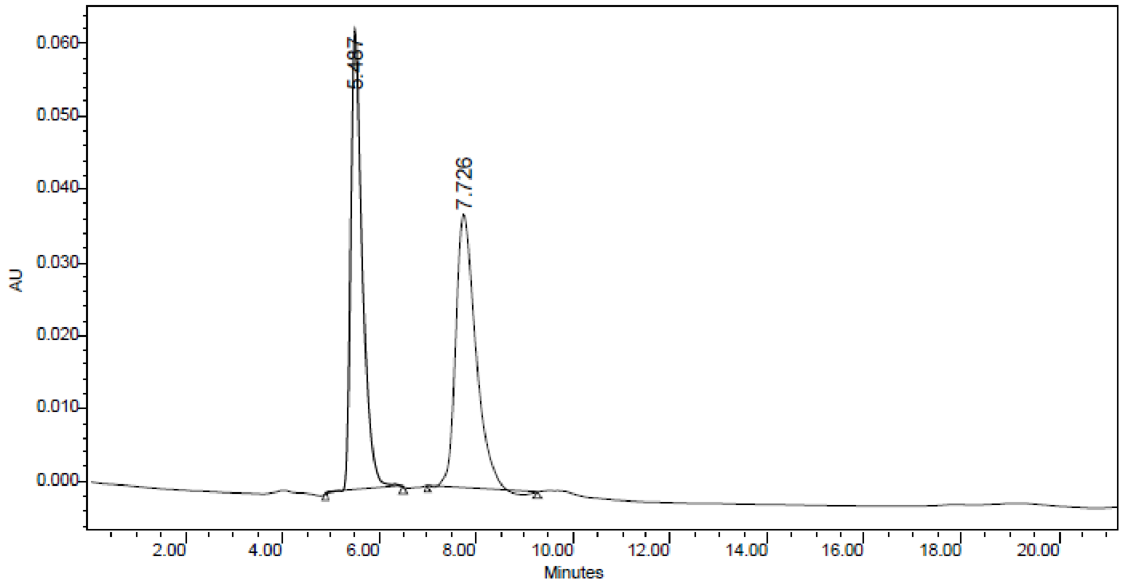

	RT (min)	Area (□V*sec)	% Area	Height (□V)	% Height
1	5.487	1063451	49.33	63214	62.78
2	7.726	1092334	50.67	37480	37.22

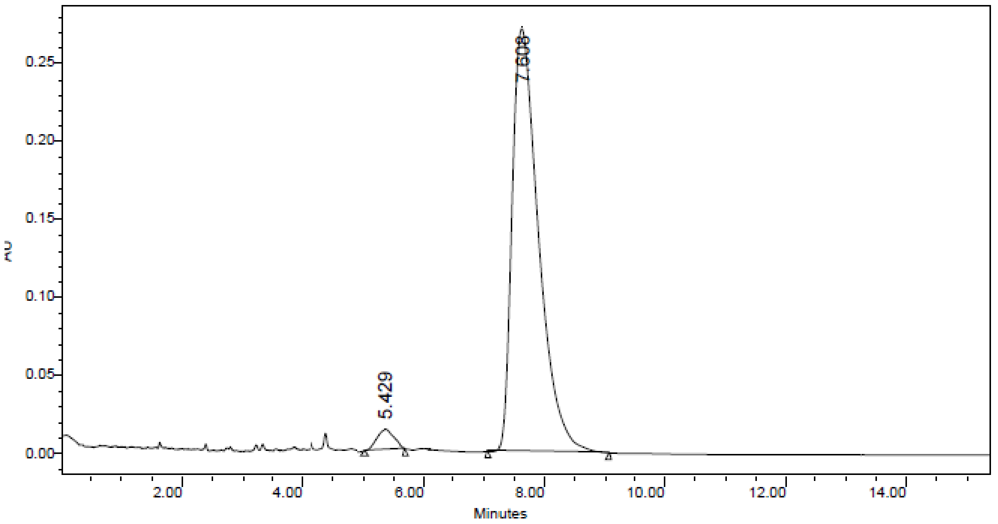

	RT (min)	Area (□V*sec)	% Area	Height (□V)	% Height
1	5.429	500691	5.69	16579	5.75
2	7.608	8300645	94.31	271948	94.25

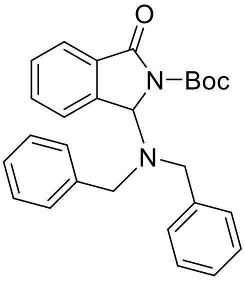

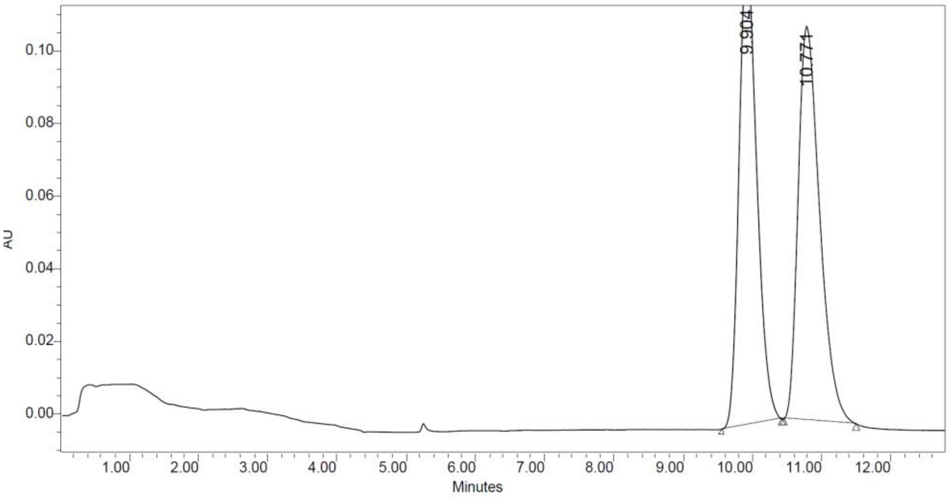

	RT (min)	Area (□V*sec)	% Area	Height (□V)	% Height
1	9.904	2305245	49.52	129328	54.42
2	10.771	2349648	50.48	108315	45.58

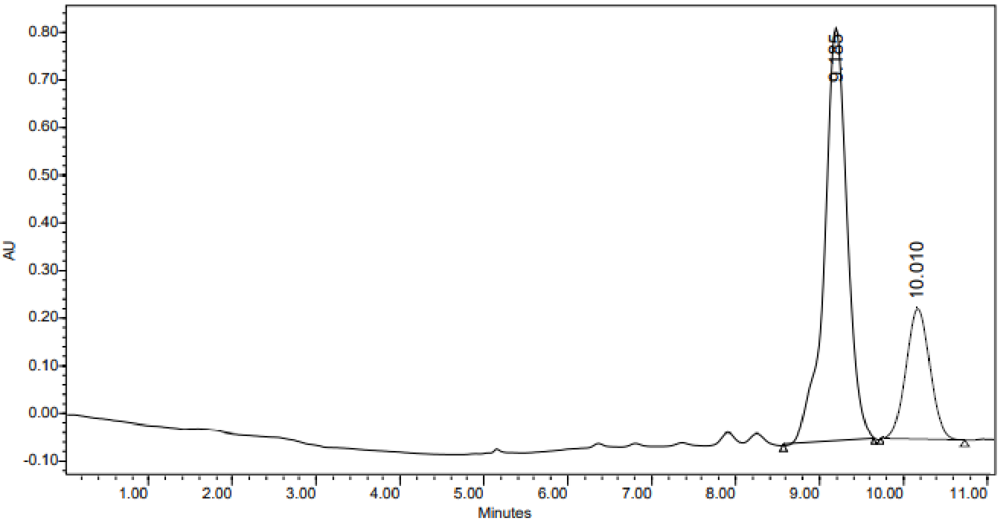

	RT (min)	Area (µV*sec)	% Area	Height (µV)	% Height
1	9.185	16466237	81.52	866698	79.16
2	10.010	3731549	18.48	228131	20.84
